# Laboratory Mice Are Frequently Colonized with *Staphylococcus aureus* and Mount a Systemic Immune Response—Note of Caution for *In vivo* Infection Experiments

**DOI:** 10.3389/fcimb.2017.00152

**Published:** 2017-05-02

**Authors:** Daniel Schulz, Dorothee Grumann, Patricia Trübe, Kathleen Pritchett-Corning, Sarah Johnson, Kevin Reppschläger, Janine Gumz, Nandakumar Sundaramoorthy, Stephan Michalik, Sabine Berg, Jens van den Brandt, Richard Fister, Stefan Monecke, Benedict Uy, Frank Schmidt, Barbara M. Bröker, Siouxsie Wiles, Silva Holtfreter

**Affiliations:** ^1^Department of Immunology, University Medicine GreifswaldGreifswald, Germany; ^2^Charles River, Research and Professional ServicesWilmington, MA, USA; ^3^Bioluminescent Superbugs Lab, Department of Molecular Medicine and Pathology, University of AucklandAuckland, New Zealand; ^4^Department of Functional Genomics, Interfaculty Institute for Genetics and Functional Genomics, ZIK FunGene, University Medicine GreifswaldGreifswald, Germany; ^5^Central Core and Research Facility of Laboratory Animals, University Medicine GreifswaldGreifswald, Germany; ^6^Alere TechnologiesJena, Germany; ^7^Institute for Medical Microbiology and Hygiene, Medical Faculty “Carl Gustav Carus”Dresden, Germany; ^8^Maurice Wilkins Centre for Molecular BiodiscoveryAuckland, New Zealand

**Keywords:** *Staphylococcus aureus*, colonization, antibody, laboratory mice, host adaptation, genotype, CC88

## Abstract

Whether mice are an appropriate model for *S. aureus* infection and vaccination studies is a matter of debate, because they are not considered as natural hosts of *S. aureus*. We previously identified a mouse-adapted *S. aureus* strain, which caused infections in laboratory mice. This raised the question whether laboratory mice are commonly colonized with *S. aureus* and whether this might impact on infection experiments. Publicly available health reports from commercial vendors revealed that *S. aureus* colonization is rather frequent, with rates as high as 21% among specific-pathogen-free mice. In animal facilities, *S. aureus* was readily transmitted from parents to offspring, which became persistently colonized. Among 99 murine *S. aureus* isolates from Charles River Laboratories half belonged to the lineage CC88 (54.5%), followed by CC15, CC5, CC188, and CC8. A comparison of human and murine *S. aureus* isolates revealed features of host adaptation. In detail, murine strains lacked *hlb*-converting phages and superantigen-encoding mobile genetic elements, and were frequently ampicillin-sensitive. Moreover, murine CC88 isolates coagulated mouse plasma faster than human CC88 isolates. Importantly, *S. aureus* colonization clearly primed the murine immune system, inducing a systemic IgG response specific for numerous *S. aureus* proteins, including several vaccine candidates. Phospholipase C emerged as a promising test antigen for monitoring *S. aureus* colonization in laboratory mice. In conclusion, laboratory mice are natural hosts of *S. aureus* and therefore, could provide better infection models than previously assumed. Pre-exposure to the bacteria is a possible confounder in *S. aureus* infection and vaccination studies and should be monitored.

## Introduction

*Staphylococcus aureus* is a dangerous opportunistic pathogen, a leading cause of bacterial infection in hospitals and in the community world-wide, and a prominent example of the crisis of antibiotic resistance (World Health Organization, 2014). Despite extensive efforts, there is currently no vaccine available (Fowler and Proctor, 2014). Thus, novel approaches for the prevention and treatment of infections are urgently required. Mice are the most commonly used surrogate host to model *S. aureus* infection with the obvious advantages of a well-characterized immune system, the wide availability of well-characterized gene knock-out strains and of being relatively easy and inexpensive to breed. However, whether mice are appropriate has often been questioned because there is broad consensus in the research community that mice are not natural hosts of *S. aureus* (Cuny et al., 2010; McCarthy and Lindsay, 2010; Capparelli et al., 2011; Mulcahy et al., 2012). Moreover, experimental colonization of mice with *S. aureus* is usually transient, and very high infection doses are routinely required (Kiser et al., 1999).

Reports on natural *S. aureus* colonization or infections in laboratory mice are scarce (Baker, 2003; Pritchett-Corning et al., 2009). We have reported an outbreak of *S. aureus* infections in C57BL/6J mice bred in a university-associated animal facility (Holtfreter et al., 2013). Male mice suffered from preputial gland adenitis (PGA), which is the most common location for abscesses in mice (Baker, 2003). The causative strain, called JSNZ, belongs to CC88, a lineage rarely found among human and animal isolates (Gorwitz et al., 2008; Ghebremedhin et al., 2009; Zhang et al., 2009; Holtfreter et al., 2016). This suggests that CC88 is a unique laboratory mouse-associated *S. aureus* lineage.

Adaptation to new hosts is a complex process, involving the loss and/or acquisition of mobile genetic elements (MGEs), such as phages, plasmids, and pathogenicity islands, as well as the accumulation of mutations in virulence genes resulting in host-specific allelic variants or loss of function (Herron-Olson et al., 2007; Guinane et al., 2010). The most prominent example for host adaptation are *hlb*-integrating Sa3int phages, which encode the human-specific immune evasion gene cluster (IEC) carrying staphylokinase (*sak*), staphylococcal complement inhibitor (*scn*) and chemotaxis inhibitory protein of *S. aureus* (CHIPS; *chp*) as well as staphylococcal enterotoxins A or P (sea, *sep*) (van Wamel et al., 2006; Sung et al., 2008). These phages are common in human isolates, but frequently lacking in animal-adapted strains, including the mouse-adapted strain JSNZ (Sung et al., 2008; Holtfreter et al., 2013).

Laboratory animal vendors generally produce mice to two different microbiological quality levels. Specific-pathogen-free (SPF) mice are free of infectious agents that are known to cause illness in mice, interfere with research, and/or are zoonotic (Mahler Convenor et al., 2014). Specific and opportunistic pathogen free (SOPF) mice are maintained free of additional microbial agents. Importantly, *S. aureus* is considered an opportunistic pathogen in mice and is, therefore, not routinely excluded from SPF barrier rooms. Since most laboratories use SPF mice for their experiments, natural *S. aureus* colonization of experimental mice might be far more common than expected. Since previous exposure of mice to *S. aureus* may influence the results of experimental infection or vaccination, it is important to learn more about *S. aureus* colonization in laboratory mice.

In this study, we analyzed the prevalence of *S. aureus* in SPF mice from all major global vendors, determined whether murine *S. aureus* isolates are adapted to their murine host, and investigated if naturally colonized mice are primed against *S. aureus*.

## Materials and methods

### Health reports of laboratory mice

Health reports were obtained from the official websites of Charles River (http://www.criver.com/products-services/basic-research/health-reports/), The Jackson Laboratory (https://www.jax.org/jax-mice-and-services/customer-support/customer-service/animal-health/health-status-reports), Taconic (http://www.taconic.com/quality/health-reports/), Janvier Labs (http://www.janvier-labs.com/rodent-research-models-services/research-models.html), Envigo (http://www.envigo.com/products-services/research-models-services/resources/health-monitoring-reports/) in November 2016.

Standard barrier production colonies (SPF) are designated by the various providers as follows: specific-pathogen-free (Charles River Europe, Janvier), VAF® (virus/antibody free; Charles River, North America), Standard (Jackson, so-called Production, Repository, and Breeding Services facilities), Low barrier [Jackson, so-called Research animal facility (RAF)], Murine Pathogen Free (Taconic), and barrier (Envigo). Specific and opportunistic pathogen free (SOPF) colonies are designated as follows: SOPF (Charles River Europe, Janvier), Elite® (Charles River, North America), intermediate barrier and high barrier (Jackson), Excluded Flora/Restricted Flora (Taconic), and Isolator (Envigo). For reasons of clarity, we will subsequently refer to the barrier types as SPF, and SOPF (Table 1).

**Table 1:** **Percentage of ***S. aureus***-positive mice and barriers in different breeding facilities, sorted by vendor and barrier status (cumulative data extracted from publicly available health reports in November 2016)**.

**Vendor**	**Barrier status^a^**	**% *S. aureus* positive mice (absolute no.)^b^**	**% *S. aureus* positive barriers (absolute no.)^b^**
Charles River, Europe	SPF	ND^c^	ND^c^
	SOPF	0.0 (0/6,766)	0.0 (0/22)
	Immunodeficient	0.0 (0/51)	0.0 (0/2)
Charles River, US and CAN	VAF	20.9 (360/1,724)	65.2 (15/23)
	SOPF (ELITE)	0.0 (0/2,524)	0.0 (0/7)
	Immunodeficient	0.0 (0/11,565)	0.0 (0/5)
The Jackson laboratory, US	Standard	0.8 (15/1,828)	9.0 (1/11)
	High	0.0 (0/1,094)	0.0 (0/6)
	Maximum	1.0 (160/16,140)^d^	13.8 (4/29)
	Low barrier (RAF)	0 (0/894)	0.0 (0/21)
	Intermediate barrier (RAF)	0 (0/321)	0.0 (0/7)
	Elevated barrier (RAF)	0 (0/60)	0.0 (0/1)
Taconic, Europe	Murine pathogen free™	8.5 (42/494)	25.0 (2/8)
	Excluded flora and restricted flora™	0.0 (0/1,648)	0.0 (0/7)
Taconic, US	Murine pathogen free™	9.7 (260/2,686)	38.5 (10/26)
	Excluded Flora and restricted Flora™	1.9 (29/1,537)	5.9 (1/17)
	Defined Flora	0.0 (0/110)	0.0 (0/4)
	Germ free	0.0 (0/71)	0.0 (0/2)
Janvier, Europe	SPF	ND^c^	ND^c^
	SOPF	0.0 (0/303)	0.0 (0/6)
Envigo (formerly Harlan), Europe	Barrier	ND^c^	ND^c^
	Isolator, immunocompetent mice	0.0 (0/610)	0.0 (0/13)
	Isolator, immunodeficient mice	0.0 (0/2,128)	0.0 (0/56)
Envigo (formerly Harlan), US	Barrier	6.0 (46/761)	87.5 (7/8)
	Isolator, immunocompetent mice	0.0 (0/85)	0.0 (0/3)
	Isolator, immunodeficient mice	0.0 (0/2,189)	0.0 (0/17)

a *Standard barrier production colonies (SPF) are designated by the various providers as follows: specific-pathogen-free (Charles River Europe, Janvier), VAF® (virus/antibody free; Charles River, North America), Standard (Jackson, so-called Production, Repository and Breeding Services facilities), Low barrier [Jackson, so-called Research animal facility (RAF)], Murine Pathogen Free (Taconic), and barrier (Envigo). Specific and opportunistic pathogen free (SOPF) colonies are designated as follows: SOPF (Charles River Europe, Janvier), Elite® (Charles River, North America), intermediate barrier and high barrier (Jackson), Excluded Flora/Restricted Flora (Taconic), and Isolator (Envigo). Each vendor has specific definitions of its barriers. For details refer to the vendor's webside*.

b *Cumulative data comprising the last 18 months (Charles River, Janvier, and Envigo), 12 months (The Jackson laboratories) or 6 months (Taconic). Envigo reported the number of positive isolators (not barriers)*.

c *S. aureus not reported based on FELASA recommendations (2002 and 2014)*.

d *According to the company, a test and cull effort was initiated to eliminate S. aureus from this barrier. This investigation has been completed, and no additional positives have been found*.

Health reports were browsed for the reported cumulative data covering the last 6 (Taconic), 12 (Jackson) or 18 months (Charles River, Envigo, and Janvier). All these vendors perform bacteriological screenings every 4 weeks (Charles River Europe, Charles River US—SOPF barriers), 6-weeks (The Jackson laboratory (Production, Repository and Breeding Services facilities), Janvier), 2 months (Envigo Europe) or quarterly (Charles River US—SPF barriers, The Jackson laboratory (Research animal facility), Taconic, Envigo US), and publish their health reports on their websites.

### *S. aureus* isolates

Murine *S. aureus* strain collection (CR strains): *S. aureus* isolates were provided by Charles River's Research Animal Diagnostic Services (Wilmington, USA). This laboratory receives more than 30,000 animals for necropsy each year from external (non-Charles River) sources. Compiled data include the sender of the isolate (pharmaceutical industry, academia, and vendors), year, country, species, and isolation site (Table 2). A total of 99 *S. aureus* strains isolated from laboratory mice were analyzed. The majority of these strains (*n* = 92) was obtained from microbiological samples sent to Charles River's Research Animal Diagnostic Services in 2004 (*n* = 20) and 2011/2012 (*n* = 71). An additional 8 isolates were collected from C57BL/6 mice during a study on the epidemiology of PGA in laboratory mice at Charles River, Raleigh, USA, in 2004.

**Table 2:** **Genotype, virulence genes, phage patterns, and ampicillin resistance of ***S. aureus*** isolates from laboratory mice**.

***S. aureus* strain**	**Year**	**Mouse strain**	**Facility^a^**	**Sample type**	**Country**	***spa* type**	**deduced MLST CC^b^**	**MGE-encoded SAgs**	***egc* SAgs**	***agr***	***eta***	***etd***	***pvl***	***mecA***	***Sa1int***	***Sa2int***	***Sa3int***	***Sa4int***	***Sa5int***	***Sa6int***	***Sa7int***	***sea***	***sep***	***sak***	***chp***	***scn***	**AmpR**
CR072	2011	Nude sent.	P	Other	USA	t11283	CC5	−	*g i m n o*	*2*	−	−	−	−	−	−	−	−	−	−	−	−	−	−	−	−	+
CR053	2011	outbred	U	col	USA	t6365	CC5	*p*	*g i m n o*	*2*	−	−	−	−	−	−	+	−	−	−	−	−	+	+	−	+	−
CR059	2011	outbred	U	col	USA	t6365	CC5	−	*g i m n o*	*2*	−	−	−	−	−	−	−	−	−	−	−	−	−	−	−	−	−
CR062	2011	outbred	U	col	USA	t6365	CC5	−	*g i m n o*	*2*	−	−	−	−	−	−	−	−	−	−	−	−	−	−	−	−	−
CR066	2011	outbred	U	col	USA	t6365	CC5	*p*	*g i m n o*	*2*	−	−	−	−	−	−	+	−	−	−	−	−	+	+	−	+	−
CR069	2011	outbred	U	col	USA	t6365	CC5	−	*g i m n o*	*2*	−	−	−	−	−	−	−	−	−	−	−	−	−	−	−	−	−
CR075	2011	outbred	U	col	USA	t6365	CC5	*p*	*g i m n o*	*2*	−	−	−	−	−	−	+	−	−	−	−	−	+	+	−	+	−
CR091	2011	outbred	U	col	USA	t6365	CC5	*p*	*g i m n o*	*2*	−	−	−	−	−	−	+	−	−	−	−	−	+	+	−	+	−
CR096	2011	CD1	V	col	USA	t6365	CC5	*p*	*g i m n o*	*2*	−	−	−	−	−	−	+	−	−	−	−	−	+	+	−	+	−
CR098	2011	CD1	U	col	USA	t6365	CC5	−	*g i m n o*	*2*	−	−	−	−	−	−	−	−	−	−	−	−	−	−	−	−	−
CR040	2011	BALB/c	V	col	Japan	t1767	CC8	−	−	*1*	−	−	−	−	−	−	−	−	−	−	−	−	−	−	−	−	−
CR014	2011	CD1	V	col	USA	t701	CC8	−	−	*1*	−	−	−	−	−	−	+	−	−	−	−	−	−	+	−	+	+
CR015	2011	CD1	V	col	USA	t701	CC8	−	−	*1*	−	−	−	−	−	−	+	−	−	−	−	−	−	+	−	+	+
CR063	2011	CD1	U	col	USA	t701	CC8	−	−	*1*	−	−	−	−	−	−	+	−	−	−	−	−	−	+	−	+	−
CR180	2012	C57BL/6	V	col	USA	neg	CC15^c^	−	−	*2*	−	−	−	−	−	+	−	−	−	−	−	−	−	−	+	+	+
CR025	2011	Swiss Web.	V	col	USA	t084	CC15	−	−	*2*	−	−	−	−	−	−	−	−	−	−	−	−	−	−	+	+	+
CR035	2011	Nude sent.	P	col	USA	t084	CC15	−	−	*2*	−	−	−	−	−	−	−	−	−	−	−	−	−	−	+	+	+
CR085	2011	Nude sent.	P	col	USA	t084	CC15	−	−	*2*	−	−	−	−	+	−	−	−	−	−	−	−	−	−	+	+	+
CR171	2012	C57BL/6	V	col	USA	t084	CC15	−	−	*2*	−	−	−	−	−	−	−	−	−	−	−	−	−	−	+	+	+
CR172	2012	C57BL/6	V	Other	USA	t084	CC15	−	−	*2*	−	−	−	−	−	+	−	−	−	−	−	−	−	−	+	+	−
CR173	2012	Swiss Web.	V	col	USA	t084	CC15	−	−	*2*	−	−	−	−	+	−	−	−	−	−	−	−	−	−	+	+	+
CR184	2012	C57BL/6	V	Other	USA	t084	CC15	−	−	*2*	−	−	−	−	−	+	−	−	−	−	−	−	−	−	+	+	−
CR102	2011	BALB/c	U	col	Canada	t094	CC15	−	−	*2*	−	−	−	−	+	−	−	−	−	−	−	−	−	−	+	+	+
CR046	2011	BALB/c	U	col	GB	t11287	CC15	−	−	*2*	−	−	−	−	+	−	−	−	−	−	−	−	−	−	+	+	−
CR057	2011	outbred	U	col	Canada	t11287	CC15	−	−	*2*	−	−	−	−	+	−	−	−	−	−	−	−	−	−	+	+	−
CR011	2011	Nude sent.	U	col	USA	t1877	CC15	−	−	*2*	−	−	−	−	−	+	−	−	−	−	−	−	−	−	+	+	−
CR179	2012	C57BL/6	V	col	USA	t1877	CC15	−	−	*2*	−	−	−	−	−	+	−	−	−	−	−	−	−	−	+	+	−
CR185	2012	C57BL/6	V	col	USA	t368	CC15	−	−	*2*	−	−	−	−	−	+	−	−	−	−	−	−	−	−	+	+	+
CR174	2012	C57BL/6	V	col	USA	t491	CC15	−	−	*2*	−	−	−	−	−	+	−	−	−	−	−	−	−	−	+	+	+
CR175	2012	C57BL/6	V	col	USA	t491	CC15	−	−	*2*	−	−	−	−	−	−	−	−	−	−	−	−	−	−	+	+	−
CR170	2012	Other	V	col	USA	t6907	CC15	−	−	*2*	−	−	−	−	−	−	−	−	−	−	−	−	−	−	+	+	−
CR167	2012	C57BL/6	V	col	USA	t774	CC15	−	−	*2*	−	−	−	−	−	+	−	−	−	−	−	−	−	−	+	+	+
CR026	2011	BALB/c	V	col	USA	t081	CC25	−	*g i m n o*	*1*	−	+	−	−	−	−	−	−	−	−	−	−	−	−	−	−	−
CR084	2011	Outbred	P	col	USA	t081	CC25	−	*g i m n o*	*1*	−	+	−	−	−	−	−	−	−	−	−	−	−	−	−	−	−
CR045	2011	Nude sent.	P	col	USA	t018	CC30	*a tst*	*g i m n o u*	*3*	−	−	−	−	−	+	+	−	+	−	−	+	−	−	+	+	+
CR064	2011	Outbred	P	col	USA	t018	CC30	*a tst*	*g i m n o u*	*3*	−	−	−	−	−	+	+	−	+	−	−	+	−	+	+	+	+
CR074	2011	Outbred	U	col	USA	t018	CC30	*a tst*	*g i m n o u*	*3*	−	−	−	−	−	+	+	−	+	−	−	+	−	−	+	+	+
CR019	2011	Other	V	col	USA	t3092	CC72	*c l*	*g i m n o*	*1*	−	−	−	−	−	−	+	−	−	−	−	−	−	+	+	+	+
CR052	2011	Outbred	U	col	USA	t11285	CC88	−	−	*3*	−	−	−	−	−	−	−	−	−	−	−	−	−	−	−	−	−
CR103	2011	Outbred	U	col	USA	t11285	CC88	−	−	*3*	−	−	−	−	−	−	−	−	−	−	−	−	−	−	−	−	−
CR134	2004	C57BL/6	P	col	USA	t12341	CC88	−	−	*3*	−	−	−	−	+	−	−	−	−	−	−	−	−	−	−	−	−
CR138	2004	C57BL/6	P	col	USA	t12341	CC88	−	−	*3*	−	−	−	−	+	−	−	−	−	−	−	−	−	−	−	−	−
CR154	2004	C57BL/6	P	col	USA	t12341	CC88	−	−	*3*	−	−	−	−	+	−	−	−	−	−	−	−	−	−	−	−	−
CR018	2011	Outbred	U	col	USA	t186	CC88	−	−	*3*	−	−	−	−	−	−	−	−	−	−	−	−	−	−	−	−	−
CR034	2011	Outbred	U	col	USA	t186	CC88	−	−	*3*	−	−	−	−	−	−	−	−	−	−	+	−	−	−	−	−	−
CR039	2011	Outbred	U	col	USA	t186	CC88	−	−	*3*	−	−	−	−	−	−	−	−	−	−	+	−	−	−	−	−	−
CR047	2011	Outbred	U	col	USA	t186	CC88	−	−	*3*	−	−	−	−	−	−	−	−	−	−	+	−	−	−	−	−	−
CR049	2011	Outbred	P	col	USA	t186	CC88	−	−	*3*	−	−	−	−	−	−	+	−	−	−	−	−	−	+	−	+	−
CR051	2011	Outbred	P	col	USA	t186	CC88	−	−	*3*	−	−	−	−	−	−	+	−	−	−	−	−	−	+	−	+	−
CR055	2011	Outbred	U	col	USA	t186	CC88^d^	−	−	*3*	−	−	−	−	−	−	−	−	−	−	+	−	−	−	−	−	−
CR061	2011	Outbred	P	col	USA	t186	CC88	−	−	*3*	−	−	−	−	−	−	+	−	−	−	−	−	−	+	−	+	−
CR065	2011	Nude sent.	P	col	USA	t186	CC88	−	−	*3*	−	−	−	−	+	−	−	−	−	−	−	−	−	−	−	−	−
CR077	2011	Swiss Web.	P	col	USA	t186	CC88	−	−	*3*	−	−	−	−	−	−	+	−	−	−	−	−	−	+	−	+	−
CR079	2011	Outbred	P	col	USA	t186	CC88	−	−	*3*	−	−	−	−	−	−	+	−	−	−	−	−	−	+	−	+	−
CR092	2011	Outbred	U	col	USA	t186	CC88	−	−	*3*	−	−	−	−	−	−	−	−	−	−	−	−	−	−	−	−	−
CR100	2011	Outbred	P	col	Japan	t186	CC88	−	−	*3*	−	−	−	−	−	−	+	−	−	−	−	−	−	+	−	+	−
CR101	2011	Other	V	col	USA	t186	CC88	−	−	*3*	−	−	−	−	+	−	−	−	−	−	−	−	−	−	−	−	−
CR117	2004	C57BL/6	V	PGA	USA	t186	CC88	−	−	*3*	−	−	−	−	−	−	−	−	−	−	−	−	−	−	−	−	−
CR118	2004	C57BL/6	V	col	USA	t186	CC88	−	−	*3*	−	−	−	−	−	−	−	−	−	−	−	−	−	−	−	−	−
CR119	2004	C57BL/6	V	col	USA	t186	CC88	−	−	*3*	−	−	−	−	−	−	−	−	−	−	−	−	−	−	−	−	−
CR120	2004	C57BL/6	V	col	USA	t186	CC88	−	−	*3*	−	−	−	−	−	−	−	−	−	−	−	−	−	−	−	−	−
CR127	2004	C57BL/6	V	PGA	Canada	t186	CC88	−	−	*3*	−	−	−	−	−	−	−	−	−	+	−	−	−	−	−	−	−
CR130	2004	C57BL/6	V	PGA	USA	t186	CC88	−	−	*3*	−	−	−	−	+	−	−	−	−	−	−	−	−	−	−	−	−
CR133	2004	C57BL/6	V	col	Canada	t186	CC88	−	−	*3*	−	−	−	−	−	−	−	−	−	+	−	−	−	−	−	−	−
CR139	2004	C57BL/6	V	PGA	USA	t186	CC88	−	−	*3*	−	−	−	−	−	−	−	−	−	−	−	−	−	−	−	−	−
CR153	2004	Other	P	PGA	USA	t186	CC88	−	−	*3*	−	−	−	−	−	−	−	−	−	−	−	−	−	−	−	−	−
CR155	2004	C57BL/6	P	PGA	USA	t186	CC88	−	−	*3*	−	−	−	−	−	−	−	−	−	−	−	−	−	−	−	−	−
CR158	2004	C57BL/6	V	col	USA	t186	CC88	−	−	*3*	−	−	−	−	−	−	−	−	−	+	−	−	−	−	−	−	−
CR169	2012	Swiss Web.	P	col	USA	t186	CC88	−	−	*3*	−	−	−	−	−	+	−	−	−	−	−	−	−	−	−	−	−
CR058	2011	Other	U	col	USA	t2085	CC88^d^	−	−	*3*	−	−	−	−	−	−	−	−	−	−	−	−	−	−	−	−	−
CR081	2011	CD1	P	col	USA	t2253	CC88	−	−	*3*	−	−	−	−	−	−	−	−	−	−	−	−	−	−	−	−	−
CR036	2011	Outbred	U	col	USA	t2649	CC88	−	−	*3*	−	−	−	−	−	−	−	−	−	−	+	−	−	−	−	−	−
CR121	2004	C57BL/6	V	col	USA	t2815	CC88	−	−	*3*	−	−	−	−	−	−	−	−	−	−	−	−	−	−	−	−	−
CR122	2004	C57BL/6	V	col	USA	t2815	CC88	−	−	*3*	−	−	−	−	−	−	−	−	−	−	−	−	−	−	−	−	−
CR140	2004	C57BL/6	V	PGA	USA	t2815	CC88	−	−	*3*	−	−	−	−	−	−	−	−	−	−	−	−	−	−	−	−	−
CR159	2004	C57BL/6	V	col	USA	t2815	CC88	−	−	*3*	−	−	−	−	−	−	−	−	−	−	−	−	−	−	−	−	−
CR050	2011	Outbred	P	col	USA	t325	CC88	−	−	*3*	−	−	−	−	−	−	+	−	−	−	−	−	−	+	−	+	−
CR013	2011	Outbred	U	col	USA	t448	CC88	−	−	*3*	−	−	−	−	−	−	−	−	−	−	−	−	−	−	−	−	−
CR094	2011	Outbred	U	col	USA	t448	CC88	−	−	*3*	−	−	−	−	−	−	−	−	−	−	−	−	−	−	−	−	−
CR097	2011	Outbred	U	col	USA	t448	CC88	−	−	*3*	−	−	−	−	−	−	−	−	−	−	−	−	−	−	−	−	−
CR030	2011	Outbred	U	col	USA	t5562	CC88	−	−	*3*	−	−	−	−	−	−	−	−	−	−	−	−	−	−	−	−	−
CR087	2011	Outbred	P	col	USA	t690	CC88	−	−	*3*	−	−	−	−	−	−	−	−	−	−	−	−	−	−	−	−	−
CR093	2011	Outbred	P	col	USA	t690	CC88	−	−	*3*	−	−	−	−	−	−	−	−	−	−	−	−	−	−	−	−	−
CR143	2004	C57BL/6	V	PGA	Canada	t690	CC88	−	−	*3*	−	−	−	−	−	−	−	−	−	+	−	−	−	−	−	−	−
CR007	2011	Outbred	P	col	USA	t7558	CC88	−	−	*3*	−	−	−	−	−	−	−	−	−	−	−	−	−	−	−	−	−
CR009	2011	Other	V	col	USA	t786	CC88	−	−	*3*	−	−	−	−	−	−	−	−	−	−	−	−	−	−	−	−	−
CR022	2011	Outbred	U	col	USA	t786	CC88	−	−	*3*	−	−	−	−	−	−	−	−	−	−	−	−	−	−	−	−	−
CR106	2011	Nude sent.	P	col	USA	t786	CC88	−	−	*3*	−	−	−	−	−	−	−	−	−	−	−	−	−	−	−	−	−
CR123	2004	C57BL/6	V	col	USA	t786	CC88	−	−	*3*	−	−	−	−	+	−	−	−	−	−	−	−	−	−	−	−	−
CR129	2004	C57BL/6	V	col	USA	t786	CC88	−	−	*3*	−	−	−	−	+	−	−	−	−	−	−	−	−	−	−	−	−
CR141	2004	C57BL/6	V	PGA	USA	t786	CC88	−	−	*3*	−	−	−	−	+	−	−	−	−	−	−	−	−	−	−	−	−
CR145	2004	C57BL/6	V	PGA	USA	t786	CC88	−	−	*3*	−	−	−	−	−	−	−	−	−	−	−	−	−	−	−	−	−
CR006	2011	BALB/c	P	col	USA	t056	CC101	−	−	*1*	−	−	−	−	−	−	+	−	−	−	−	−	−	+	−	+	−
CR044	2011	Outbred	P	col	USA	t189	CC188	−	−	*1*	−	−	−	−	−	−	+	−	−	−	−	−	−	+	−	+	+
CR125	2004	C57BL/6	V	col	Canada	t189	CC188	*p*	−	*1*	−	−	−	−	−	−	+	−	−	−	−	−	+	+	−	+	+
CR132	2004	C57BL/6	V	PGA	USA	t189	CC188	*p*	−	*1*	−	−	−	−	−	−	+	−	−	−	−	−	+	+	−	+	−
CR144	2004	C57BL/6	V	col	USA	t189	CC188	−	−	*1*	−	−	−	−	−	−	+	−	−	−	−	−	−	+	−	+	+
CR147	2004	C57BL/6	V	PGA	Canada	t189	CC188	*p*	−	*1*	−	−	−	−	−	−	+	−	−	−	−	−	+	+	−	+	+
CR148	2004	C57BL/6	V	PGA	USA	t189	CC188	*p*	−	*1*	−	−	−	−	−	−	+	−	−	−	−	−	+	+	−	+	+

a *Animal facilities were defined as university (U), vendors (V) and pharmaceutical industry (P)*.

b *spa types were clustered by BURP analysis into CCs and corresponding MLST CCs were deduced using the Ridom database*.

c *MLST typing results: ST15*.

d *MLST typing results: ST88*.

*Nude sent., Nude sentinels; Swiss Web., Swiss Webster; col, colonization (nasopharyngeal sample); PGA, preputial gland adenitis (pus sample); agr, accessory gene regulator; Staphylococcal enterotoxins (SEs) are indicated by single letters (a = sea, etc.). tst, toxic shock syndrome toxin 1 gene; egc, superantigen genes of the enterotoxin gene cluster, i.e. seg, sei, sem, sen, seo, and seu; eta/etd, exfoliative toxins a and d; luk−PV, Panton−Valentine leukocidine gene; Sa1int, S. aureus integrase type 1; sak, Staphylokinase gene; chp, gene encoding the chemotaxis inhibitory protein; scn, staphylococcal complement inhibitor gene; AmpR, ampicillin resistance*.

Overall, most *S. aureus* strains were isolated from specimens provided by pharmaceutical and biotechnology industries (*n* = 27), academia (*n* = 29), and other vendors (*n* = 43) (Table 2). Most samples were obtained in the USA (*n* = 89), while a few isolates originating from Canada (*n* = 7), Japan (*n* = 2) and Great Britain (*n* = 1) (Table 2). The majority of isolates were obtained from the nasopharynx of symptom free animals (*n* = 83), while the remaining strains were isolated from inflamed preputial glands (*n* = 13) or other body sites (vagina, lung; *n* = 3) (Table 2). To avoid a sampling bias, we included a maximum of 4 isolates from the same barrier, mouse strain and time point in the study cohort.

PGA strains and sera: Five *S. aureus* strains from colonized (nasopharynx), healthy laboratory mice and five strains from mice with preputial gland adenitis (PGA) were obtained from the Charles River breeding facilities at Kingston NY and Hollister CA, respectively. All animals were male C57BL/6NCrl mice, 7–8 weeks of age and housed under SPF conditions. Animals were selected from those destined for euthanasia, either due to surplus animal production or for their presentation with PGA. Sera were obtained via cardiac puncture of animals euthanized with inhaled CO_2_. Sera from five SOPF C57BL/6NCrl mice from Wilmington MA, which are per definition not exposed to *S. aureus*, served as negative controls.

ZSFV strains: We screened mice held under SPF conditions as well as care takers at the Central service and research facility for laboratory animals (Zentrale Service- und Forschungseinrichtung für Versuchstiere) at the University Medicine in Greifswald, Germany, for *S. aureus* colonization. Mice from all SPF barriers were systematically screened for *S. aureus* colonization by testing several stool samples from each mouse strain and each barrier room. We also obtained stool samples from parent mice and their offspring (after weaning until 16 weeks). The samples were dissolved in a small volume of PBS and then cultivated in mannitol salt medium [1.0% (w/v) BactoTM Tryptone (BD, France), 7.5% (w/v) sodium chloride (Sigma, USA), 1.0% (w/v) D-mannitol (Sigma, USA), 2.5% (w/v) yeast extract (Oxoid, UK)]. After 48 h serial dilutions were plated on BBL™ Mannitol Salt Agar (BD, USA) and identified as outlined below.

Nasal colonization of animal care takers was investigated by swabbing the nasal vestibule with a rayon swab (BBL CultureSwabTM Liquid Stuart, BD, USA) as previously reported (Holtfreter et al., 2016). Animal care takers (*n* = 13) were screened four times within 10 months in 2013–2014.

Human *S. aureus* strains: Human *S. aureus* strains (*n* = 107) were obtained from healthy blood donors in Northern Germany in 2002 (T strains, *n* = 55) and 2005–2006 (SH strains, *n* = 52). *Spa* types as well as *agr* type, superantigen gene patterns and Saint phage groups of these strains were previously reported (Holtfreter et al., 2007; Goerke et al., 2009).

Human CC88 strains: 5 human *S. aureus* isolates were obtained from a study of patients with bacteraemia admitted to hospital in Auckland as previously reported (Holtfreter et al., 2013). Another 13 strains from various sources were kindly provided by Stefan Monecke, Alere Technologies. Moreover, three CC88 isolates were obtained in the population-based studies SHIP and SHIP-TREND (Holtfreter et al., 2016), and another three isolates were kindly provided by Sebastian Stentzel, University Medicine Greifswald (Table S4).

### Ethics statement

Human plasma samples were obtained from healthy adult volunteers. All participants gave written informed consent in accordance with the Declaration of Helsinki, and the study was approved by the ethics board of the Medical Faculty of the University of Greifswald (BB 014/14; 24.01.2014).

Murine blood samples and nasopharyngeal swabs were obtained from C57BL/6 SPF and SOPF mice during routine health monitoring at Charles River facilities in Hollister CA, Kingston NY, and Wilmington MA, USA (Protocol number P06172002—Holding & Euthanasia of Animals for Diagnostic Testing and Health Monitoring). All animal work was performed in accordance with United States Public Health Service Policy on Humane Care and Use of Laboratory Animals and the US Animal Welfare Act.

### *S. aureus* identification and genotyping

*S. aureus* was identified by colony morphology on mannitol salt agar (MSA) plates, *S. aureus*–specific latex agglutination test (Staph Xtra Latex kit, ProLexTM, Richmond Hill, ON, Canada) as well as gyrase and nuclease PCR (see below). *Spa* genotyping and multilocus sequence typing (MLST) were performed as described elsewhere (Enright et al., 2000; Harmsen et al., 2003). *Spa* types were clustered into clonal lineages using the BURP algorithm of the Ridom Software, the calculated cost between members of a group being ≤ 5 (version 2.2.1). *Spa* types shorter than five were excluded from the analysis because they do not allow the reliable deduction of ancestries. MLST typing was performed on selected isolates to confirm the *spa* clustering results (CR126, t189; CR055, t186; CR111, t2815; CR105, t084), or to assign a lineage to *spa*-negative strains (CR180) and isolates with excluded *spa* types (CR058, t2085).

### Virulence gene detection

Multiplex PCRs were applied to detect a total of 25 *S. aureus* virulence genes, including gyrase (*gyr*), methicillin resistance (*mecA*), Panton-Valentine leukocidin (*pvl*), staphylococcal superantigens (*sea-selu, tst*), exfoliative toxins (*eta, etd*) and *agr* group 1–4 (Holtfreter et al., 2007; Goerke et al., 2009).

### Phage integrase gene detection

*S. aureus* bacteriophage types (*Sa1int–Sa7int*) were detected by multiplex PCR as previously reported with the following modifications (Goerke et al., 2009). PCRs were performed with the GoTaq Flexi DNA polymerase system (Promega, Mannheim, Germany). Each reaction mix (25 μl) contained 5 μl 5 × GoTaq reaction buffer, 100 μM deoxynucleoside triphosphates (dATP, dCTP, dGTP, and dTTP; Roche Diagnostics, Mannheim, Germany), 5 mM MgCl2, 320 nM of each primer, 1.0 U GoTaq® Flexi DNA polymerase and 10–20 ng of template DNA. An initial denaturation of DNA at 95°C for 5 min was followed by 25 cycles of amplification (95°C for 30 s, 55°C for 30 s and 72°C for 45 s), ending with a final extension phase at 72°C for 10 min. All PCR products were resolved by electrophoresis in 1.5% agarose gels (1 × TBE buffer), stained with RedSafe^TM^ (INtRON Biotechnology, Korea) and visualized under UV light. Positive controls included DNA from *Saint* gene-positive *S. aureus* reference strains Newman (*Sa3int, Sa5int, Sa6int, Sa7int*), MSSA476 (*Sa4int*), USA300 (*Sa2int, Sa3int*), and T132-1 (*Sa1int, Sa3int*) (Goerke et al., 2009). In addition to standard PCR controls for contamination events, *S. aureus* strain 8325-4 served as phage-negative control.

### Immune evasion cluster gene detection

The Sa3int phage-encoded immune evasion cluster (IEC) genes were detected by multiplex PCR using primers specific for *sak* (*sak*-5 ATGCTCAAAAGARGTTTATT; sak-3 TTATTTCTTTTCTATAAYAACC), *chp* (chp-5 TTAGCAACAACAGTTTTAGC; chp-3 TAAGATGATTTAGACTCTCC), *scn* (scn-5 ACTTTAGCAATCGTTTTAGC; scn-3 CTGAAATTTTTATAGTTCGC), *Sa3int* (Sa3int-5 GTTAAAGAAAATACCTACCG; Sa3int-3 TTCTTTWGCGTGTTCTTTTG) and gyrase (gyr-5 AGTACATCGTCGTATACTATATGG; gyr-3 ATCACGTAACAGTTCAAGTGTG). Each reaction mix (25 μl) contained 5 μl 5 × GoTaq® reaction buffer, 100 μM deoxynucleoside triphosphates (dATP, dCTP, dGTP, and dTTP; Roche Diagnostics, Mannheim, Germany), 5 mM MgCl2, 200 nM of scn-5, scn-3, chp-5, chp-3, gyr-5, and gyr-3, 400 nM Sa3int-5 and Sa3int-3; 600 nM sak-5, and sak-3, 1.0 U GoTaq® Flexi DNA polymerase and 10–20 ng of template DNA. An initial denaturation of DNA at 95°C for 5 min was followed by 30 cycles of amplification (95°C for 30 s, 57.5°C for 30 s and 72°C for 60 s), ending with a final extension phase at 72°C for 10 min. All PCR products were resolved by electrophoresis as described above. Positive controls included DNA from IEC gene-positive *S. aureus* reference strains [Newman (*sea, sak, chp, scn, Sa3int, gyr*) and N315 (*sep, sak, scn, Sa3int, gyr*)]. In addition to standard PCR controls for contamination events, *S. aureus* strain 8325-4 served as IEC-negative control.

### Ampicillin resistance

Ampicillin resistance was determined by patching *S. aureus* isolates onto tryptic soy agar (TSA) with 100 μg/ml Ampicillin (Sigma Aldrich, New Zealand) and determining growth after overnight incubation at 37°C. If no concordant results were obtained within three biological replicates data were labeled as ambiguous (+).

### Coagulation assay

Murine CC88 isolates (*n* = 19) and human CC88 isolates (*n* = 17) were grown for 7–8 h in in tryptic soy broth (TSB) to reach stationary phase. Sixty-five Microliters of the bacterial cultures (ca 2.5 × 10^9^ CFU/ml) were mixed with 500 μl of human or murine heparinized plasma (Equitech-Bio, Kerrville, USA) and incubated at 37°C without agitation. The coagulation state was visually examined in a blinded fashion after 2, 4, and 24 h using a modified coagulation score (Sperber et al, 1975). No coagulation was reported as 0, small coagulation flakes as 1, a medium-sized clot as 2, a large clot as 3 and complete coagulation (tube can be inverted) as 4.

### Protein a deficient *S. aureus* strains

Protein A deficiency was transferred from *S. aureus* SA113Δ*spa*:Ery to *S. aureus* JSNZ (MLST-CC88) and *S. aureus* PGA12 (MLST-CC1) by phage transduction using Phi11 as previously reported (Holtfreter et al., 2013).

### Detection of anti- *S. aureus* serum antibodies by ELISA

Murine serum IgG against *S. aureus* extracellular proteins and surface antigens was determined by ELISA. To avoid unspecific binding of serum IgG, we employed the protein A deficient JSNZ strain. JSNZ Δ*spa* was cultured in 600 ml TSB with 600 μM Bipyridyl (moderate iron limitation) in a 3l flask at 100 rpm linear shaking and 37°C and growth was monitored closely. Bacteria were harvested 3 h after entering stationary phase. Extracellular proteins were precipitated with 10% (w/v) trichloric acid as previously reported (Holtfreter et al., 2009). Bacterial cells were washed with PBS, adjusted to an OD600 nm of 1.0 (corresponds to 5 × 10^8^ CFU/ml) and UV-inactivated for 30 min. Microtiter plates were coated with 0.5 μg extracellular proteins per well or 2.5 × 10^7^ CFU UV-inactivated cells in coating buffer pH9.6 (Candor Bioscience GmbH, Wangen, Germany) over night at 4°C and afterwards blocked for 1 h at room temperature with 10% FCS in PBS. Murine sera were diluted 1:100 in blocking buffer, added to the ELISA plate and incubated for 1 h at room temperature. Due to high antibody titers, sera from infected mice from Hollister were additionally tested at 1:1000 and 1:10,000 dilutions. Anti-*S. aureus* serum IgG was quantified with a peroxidase-conjugated goat-anti-mouse detection antibody (1:20,000 in blocking buffer; Jackson ImmunoResearch, Suffolk, UK) and BD OptEIA^TM^ TMB substrate reagent. Measurements were performed in duplicates and each ELISA was performed at least twice.

### Detection of anti- *S. aureus* serum antibodies with the xMAP® technology

FlexMap technology was used to quantify mouse serum IgG binding to a total of 58 *S. aureus* proteins. Purified recombinant *S. aureus* proteins were produced in *E. coli* by Protagen AG, Dortmund, Germany (Table S6) kindly provided by S. Engelmann, Ernst Moritz Arndt University Greifswald, or generated in our group. In addition, we coupled the extracellular proteins of JSNZΔ*spa* (CC88) and PGA12Δ*spa* (CC1). Extracellular proteins were obtained by culturing both strains in 600 ml TSB with 600 μM Bipyridyl (moderate iron limitation) in a 3 l flask at 100 rpm linear shaking and 37°C. Bacterial supernatants were harvested 3.5 h after entering stationary phase and extracellular proteins were precipitated with 10% (w/v) trichloric acid as previously reported (Holtfreter et al., 2009). The purified proteins were covalently coupled to MagPlex®-beads via peptide bonds (Luminex Corporation, Austin, TX) (Stentzel et al., 2015). To validate coupling efficiency, protein coupled beads were stained with an antibody directed against the recombinant protein tag and a phycoerythrin (PE-) conjugated antibody directed against the tag antibody (Stentzel et al., 2015). All 58 MagPlex® bead entities were pooled into a beads master mix (multiplex assay). The murine serum IgG response directed against the 58 *S. aureus* proteins was analyzed with the FLEXMAP 3D® system using the xPONENT software (version 4.1). To assure assaying the sera in the linear antibody binding range for each coupled protein, all sera were measured in seven dilutions (1:50, 500, 1000, 10,000, 50,000, 100,000, and 200,000). Serum samples were diluted with assay buffer (50% PBS, 1% (w/v) Bovine serum albumin pH7.4, 50% (v/v) low cross buffer; Candor, 1000050). Afterwards, 50 μl of antigen coupled beads master mix (125 beads per well) was co-incubated with 50 μl of diluted serum samples overnight at 2–4°C on the plate shaker. The plates were washed three times with 100 μl of washing buffer (PBS, 0.05% tween-20 pH7.4). The beads were resuspended in 50 μl of R-Phycoerythrin (RPE)-conjugated goat anti-mouse IgG (final concentration 5 μg/ml; Dianova, 115-116-146), incubated for 60 min at RT in the dark on the plate shaker, washed, and finally resuspended in 100 μl of sheath fluid. Measurements were performed on the FLEXMAP 3D™ system as previously reported (Stentzel et al., 2015). The assay was performed in duplicate. All measurements were corrected for non-specific background signal by subtracting the maximum fluorescence intensities (MFIs) of sample beads (incubated with mouse sera) to MFIs of control beads (incubated with assay buffer). Next, the seven normalized MFI values were used to generate a saturation curve based model using a non-least squared algorithm as reported (Stentzel et al., 2015). The product of the half-maximal MFI and the corresponding serum dilution was calculated as this reflects the antigen binding intensity of antibodies contained in each serum sample. Values below the limit of detection were set to the lowest detected value. Calculations were performed using the R software package (R 3.0.1) (R Development Core Team, 2013).

### Statistics

Data analysis was performed using the GraphPadPrismX3 package. Antibody titers in colonized, infected and SOPF mice were not normally distributed and therefore compared with the Mann-Whitney-Test, using the Bonferoni correction for multiple testing.

## Results

### SPF mice are frequently colonized with *S. aureus*

To gain an insight into the prevalence of *S. aureus* in commercially available laboratory mice, we screened all publicly available health reports from the major vendors (Charles River, The Jackson Laboratories, Taconic, Janvier labs, and Envigo). All these vendors perform regular bacteriological screenings and publish their health reports on their websites. The highest cumulative rates were reported in SPF mice from the North American Charles River facilities (20.9%), followed by Taconic (9.7 and 8.5% in the US and Europe, respectively; Table 1). In contrast, standard colonies from The Jackson Laboratories, were almost free of *S. aureus* (0.8%). Importantly, the European branches of Janvier, Charles River, and Envigo do not report the *S. aureus* status of SPF mice as this is not recommended for immunocompetent mice in health monitoring guidelines promulgated by the Federation of Laboratory Animal Science Associations (FELASA; Mahler Convenor et al., 2014). At all vendors, SOPF barrier rooms, isolators, and areas housing immune-deficient mice are actively managed as to exclude opportunistic pathogens and were hence *S. aureus-*free.

As a standard procedure at all vendors of laboratory mice, colonies in new barrier rooms are set up with *S. aureus*-free breeders from well-monitored foundation colonies (personal communication, K. Pritchett-Corning). Therefore, *S. aureus* must be accidentally introduced into some colonies at a later stage. Retrospective data obtained from five SPF barriers of Charles River showed that it can take between 10 months and 4 years until an *S. aureus*-negative SPF barrier turns *S. aureus*-positive (Table S1). This demonstrates that introduction of *S. aureus* into a barrier is a very rare event. However, regular microbiological screenings at Charles River, US, show that once *S. aureus* is introduced into a barrier, it persists there for years (personal communication, K. Pritchett-Corning).

Overall, our findings clearly demonstrate that laboratory mice from SPF barriers are frequently colonized with *S. aureus*. Only mice monitored for *S. aureus* and from which these bacteria are deliberately excluded are guaranteed *S. aureus*-free.

### *S. aureus* is efficiently transmitted from parents to offspring

**Figure 1:**
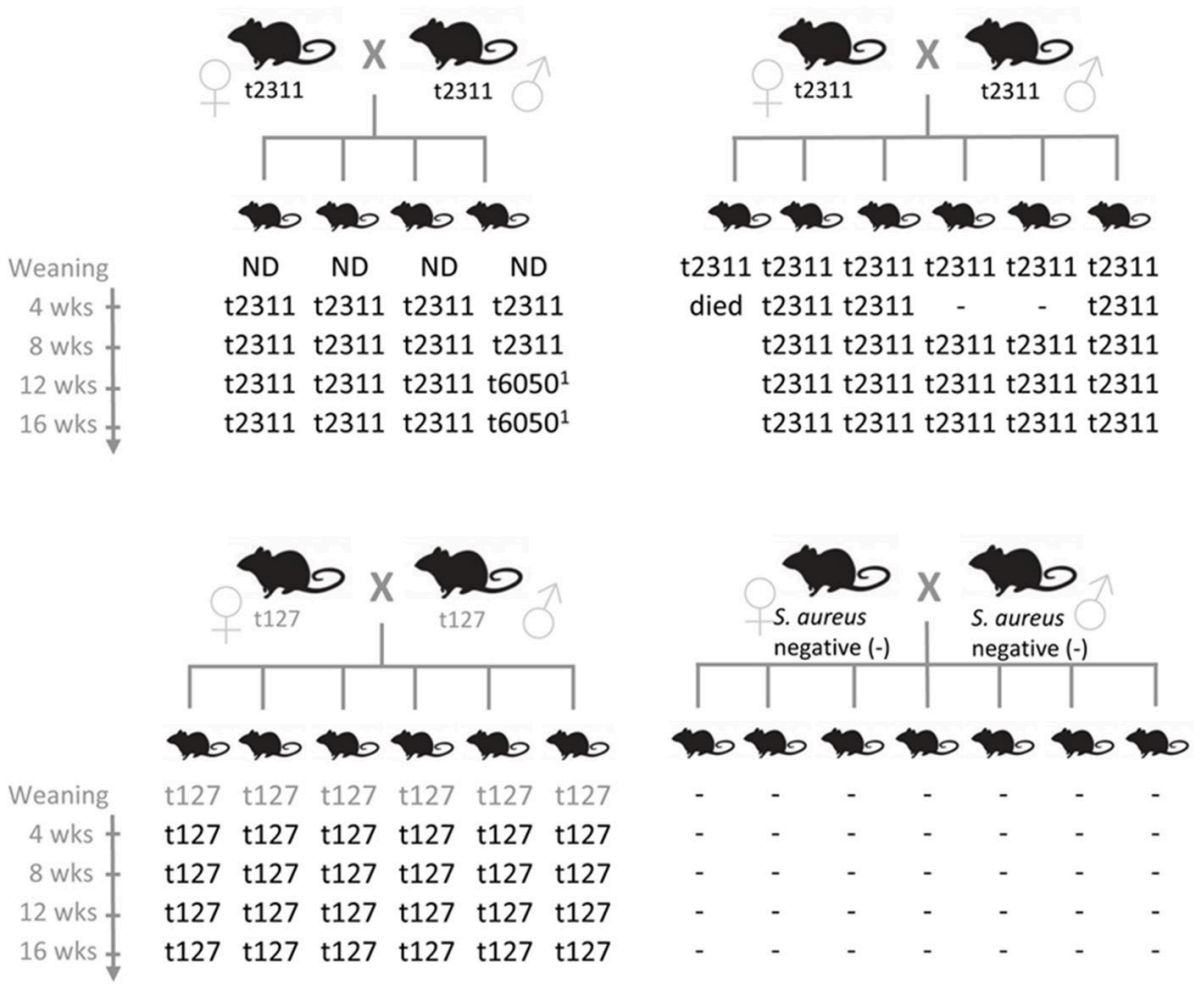
*****S. aureus*** is efficiently transmitted from murine parents to their offspring**. *S. aureus* isolates were obtained from individual stool samples of breeders and their offspring at weaning (20–23 days after birth) as well as 4, 8, 12, and 16 weeks later. Clonal relationship of the *S. aureus* isolates was determined by *spa* typing. Gray *spa* types were derived from a pooled stool sample from either parental mice or offspring. *Spa* types t2133 and t6050 are closely related (both CC88). *Spa* type t127 belongs to CC1. −, *S. aureus* negative; ND, not determined.

To elucidate the reasons for the stability of *S. aureus* colonization in breeding colonies, we examined *S. aureus* transmission among mice. One *S. aureus* negative and three *S. aureus* positive C57BL/6 breeding pairs were selected and *S. aureus* colonization of the offspring was determined at weaning as well as 4, 8, 12, and 16 weeks later. *S. aureus* was isolated from stool samples of individual mice and *spa-*typed to prove clonal origin. Notably, *S. aureus* was rapidly and very efficiently transmitted from parents to offspring. All young mice became colonized, most by weaning, and *S. aureus* then persisted in the gut of most mice throughout the period of study (Figure 1). Hence, early exposure to *S. aureus* leads to persistent colonization in laboratory mice. In contrast, the offspring of *S. aureus*-negative mice remained *S. aureus* negative throughout the whole testing period. These results suggest that once introduced into a facility, *S. aureus* will be readily transmitted to the offspring of colonized breeding pairs but not necessarily between cages.

### CC88 is the dominant *S. aureus* lineage in laboratory mice

**Figure 2:**
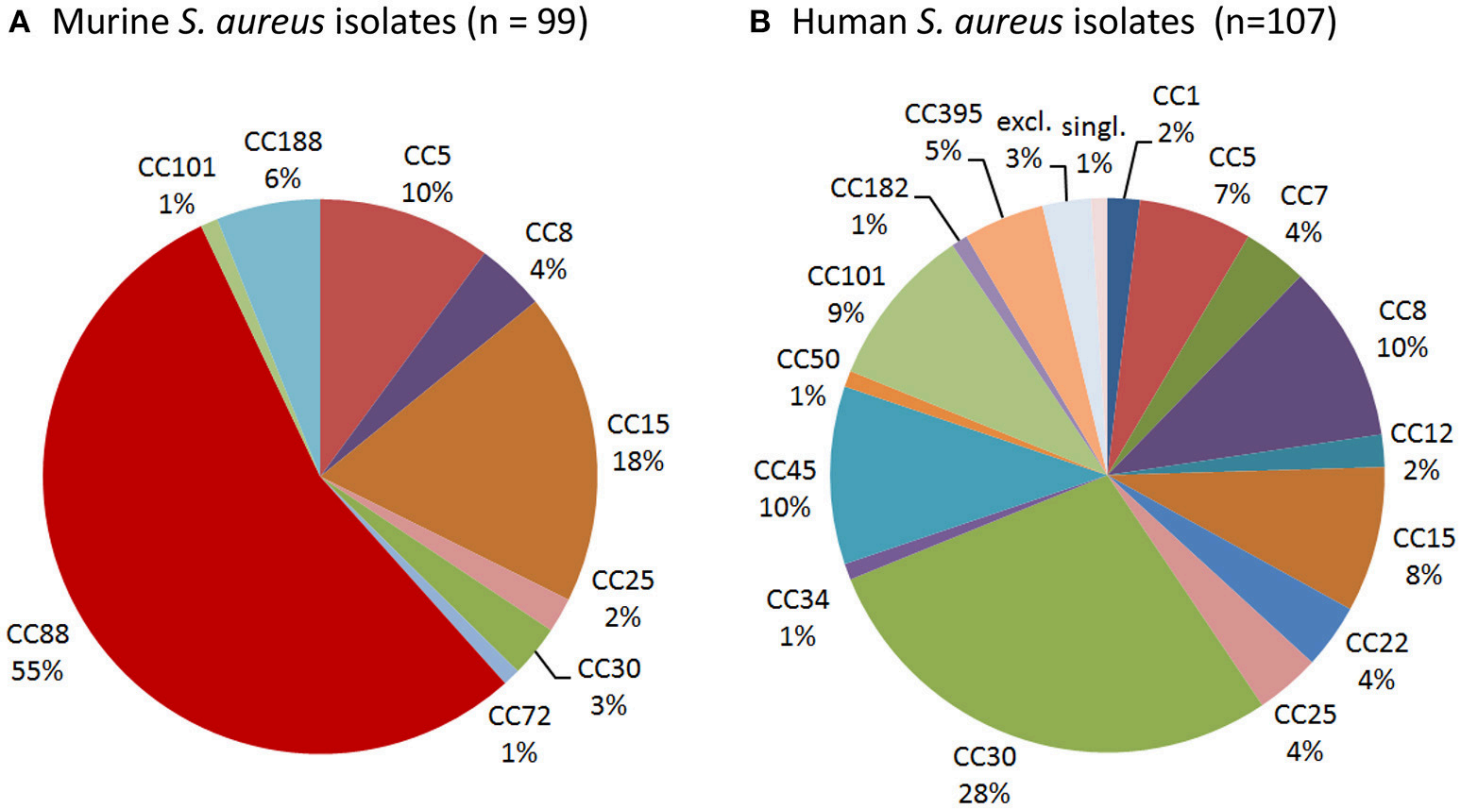
**CC88 is common among murine but not human ***S. aureus*** strains**. *Spa* genotyping was employed to resolve the population structure of murine *S. aureus* isolates **(A)** and compare it to a collection of 107 human *S. aureus* isolates **(B)**. *Spa* types were clustered into MLST-derived clonal complexes. MLST-CC clustering of the human nasal *S. aureus* isolates (*n* = 107), which has been previously published (Holtfreter et al., 2007), was refined based on the current SpaServer database. While CC88 was the dominant murine lineage, it was absent from the studied human collection.

To determine whether laboratory mice are colonized with typical human isolates or rather mouse-adapted strains such as JSNZ (Holtfreter et al., 2013), we characterized a total of 99 *S. aureus* isolates from laboratory mice. *Spa* genotyping was employed to resolve the population structure and compare it to a well-characterized collection of 107 nasal isolates from healthy *S. aureus* carriers from Northern Germany (Holtfreter et al., 2007). More than half (54/99) of the murine strains belong to the MLST-CC88 and were therefore closely related to JSNZ (Figure 2, Table 2). In contrast, CC88 were not detected among human colonizing strains (Figure 2, Table S2). Lineages that are commonly found in humans (CC5, 8, 12, 15, 25, and 30) accounted for only 37.4% (37/99) of the murine isolates (Figure 2). Moreover, the livestock-associated lineages CC72 (*n* = 1) and CC188 (*n* = 6) were detected among the murine isolates (Sung et al., 2008; McCarthy et al., 2012).

CC88 strains were already present in samples obtained in 2004, suggesting that this lineage is a long-standing companion of laboratory mice (Table 2). Moreover, CC88 strains were widely distributed as these strains were submitted by various customers (pharmaceutical industry, academia, and vendors) from the US, Canada and Japan. Apart from CC88, CC15 isolates were frequent in the sampling cohort, which also originated from various customers and several countries (US, Canada, Great Britain; Table 2). The described *S. aureus* strain spectrum was also represented in the animal facility of University Medicine Greifswald. None of the local animal care takers carried an animal clone confirming that transmission between humans and mice is rare (Table S3).

In conclusion, these data reveal that CC88, which also caused a severe outbreak of preputial gland adenitis at the animal facility in Auckland (Holtfreter et al., 2013), is the predominant lineage among murine *S. aureus* isolates from laboratory mice.

### *S. aureus* isolates from laboratory mice adapt to their murine host by eliminating MGEs encoding human-specific virulence factors

To investigate host adaptation, we screened the murine *S. aureus* isolates for bacteriophages, MGE-encoded superantigens (SAg), and ampicillin resistance. A collection of 107 nasal isolates from healthy *S. aureus* carriers (Table S2), supplemented by a total of 24 human CC88 strains from around the globe (Table S4), served as control (Holtfreter et al., 2007).

Firstly, we screened all murine strains for bacteriophages and the Sa3int phage-encoded human-specific IEC genes. The most prevalent phage families among human isolates, Sa2int and Sa3int phages, were rarely found in murine isolates. Sa2int phages were detected in 12.1% of murine isolates as compared to 33.6% of human isolates (*p* < 0.001) (Table 2, Table S2). Similarly, IEC-encoding Sa3int phages were common among human strains, but infrequently found in murine isolates (79.4 vs. 26.3%; *p* < 0.001). Unexpectedly, all murine and human CC15 isolates harbored *chp* and *scn* but lacked the *Sa3int* gene. These strains carry immobilized remnants of the *Sa3int* phage including the IEC genes *scn* and *chp* in the bacterial genome (Figure S1). As MGEs are linked to *S. aureus* clonal lineages, we also stratified the prevalence Sa3int phages by CC (Table 3). Only 12.9% (7/54) of murine CC88 strains carried IEC-encoding Sa3int phages compared to 100% (24/24) of the human CC88 isolates (*p* < 0.001).

**Table 3:** **Prevalence of IEC-encoding Sa3int phages, MGE-encoded superantigen genes and ampicillin resistance in murine and human isolates**.

		**% positive murine *S. aureus* isolates (absolute no.)**	**% positive human *S. aureus* isolates (absolute no.)**	***p*-value**
IEC-encoding Sa3int phages	CC5	50.0 (5/10)	85.7 (6/7)	n.s.
	CC8	75.0 (3/4)	81.8 (9/11)	n.s.
	CC15*^a^*	0.0 (0/18)	0.0 (0/9)	n.s.
	CC88	12.9 (7/54)	100.0 (24/24)	*P* < 0.001
	All strains	26.3 (26/99)	79.4 (104/131)	*P* < 0.001
MGE-encoded SAg genes	CC5	50.0 (5/10)	57.1 (4/7)	n.s.
	CC8	0.0 (0/4)	100.0 (11/11)	*P* < 0.001
	CC15	0.0 (0/18)	0.0 (0/9)	n.s.
	CC88	0.0 (0/54)	45.8 (11/24)	*P* < 0.001
	All strains	13.1 (13/99)	59.5 (78/131)	*P* < 0.001
Ampicillin resistance	CC5	10.0 (1/10)	85.7 (6/7)	*P* < 0.01
	CC8	50.0 (2/4)	81.8 (9/11)	*P* < 0.05
	CC15	55.6 (10/18)	77.8 (7/9)	n.s.
	CC88	0.0 (0/54)	54.0 (16/24)	*P* < 0.001
	All strains	22.2 (22/99)	66.4 (87/131)	*P* < 0.001

a *All CC15 isolates carry immobilized remnants of the Sa3int phage with most of the phage genome including the integrase missing. The IEC locus, however, is intact (see also Figure S1)*.

*IEC, immune evasion cluster encoding staphylokinase, staphylococcal complement inhibitor, chemotaxis inhibitory protein of S. aureus, as well as staphylococcal enterotoxin A or P; MGE, mobile genetic element*.

Secondly, we investigated whether murine and human strains differ in their patterns of MGE-encoded SAg genes, because these toxins act on murine T cells with much lower potency than on the human counterparts (Holtfreter and Broker, 2005). Only 13.1% (13/99) of murine *S. aureus* isolates harbored MGE-encoded SAg genes in striking contrast to 59.5% of the human isolates (78/131; *p* < 0.001; Table 3; Holtfreter et al., 2007). Mice were typically colonized either with SAg-negative lineages (CC15, CC101) or with SAg-negative variants of lineages harboring MGE-encoded SAg genes in human isolates (CC88, CC8). For example, 11/24 human CC88 strains were SAg-positive, whereas all 54 murine CC88 isolates were SAg-negative (*p* < 0.001; Tables 2, 3, Table S2).

Thirdly, all isolates were screened for ampicillin resistance, which is a common feature of human *S. aureus* strains (Holtfreter et al., 2016). Of note, only 22.2% (22/99) of the murine strains were resistant to ampicillin as compared to 66.4% of the human isolates (Table 3). Similarly, all murine CC88 strains were ampicillin sensitive, whereas 16/24 human isolates were resistant. All murine isolates were *mecA*-negative.

Overall, our findings suggest that murine *S. aureus* isolates have adapted to their murine host by eliminating MGEs encoding human-specific virulence factors, such as IEC-encoding Sa3int phages and SAg-encoding MGEs. Moreover, most murine isolates lacked ampicillin resistance.

### Murine CC88 *S. aureus* isolates coagulate mouse plasma faster than their human counterparts

**Figure 3:**
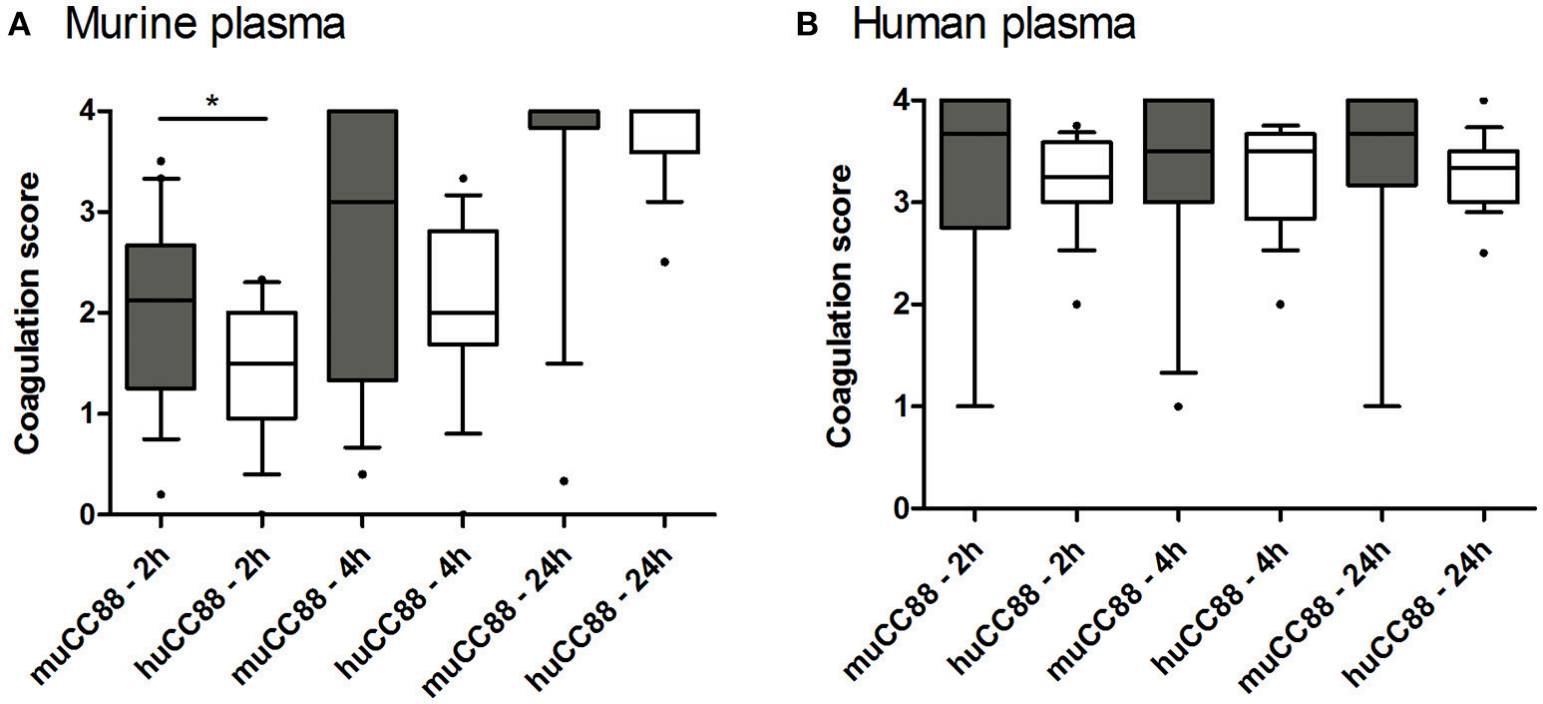
**Murine CC88 isolates coagulate murine plasma faster than human CC88 isolates**. Murine (*n* = 19) and human (*n* = 17) CC88 isolates were compared in their ability to coagulate murine **(A)** or human **(B)** plasma. 500 μl plasma were inoculated with 2.5 × 10^9^ CFU *S. aureus* and coagulation was visually assessed after 2, 4, and 24 h. At least three technical replicates were performed for each sample, and the average values are depicted as box plots. Boxes indicate the median as well as the 25–75 percentiles; whiskers illustrate the 10–90 percentiles. Statistics: Mann Whitney *U*-test. Key: muCC88, murine CC88 strains; huCC88, human CC88 strains. ^*^p ≤ 0.05.

*S. aureus* exploits the host's coagulation system by hiding itself from host immune cells in a dense fibrin network. These bacteria produce several immune modulatory factors that can shift haemostasis toward coagulation (coagulase, van Willebrandt factor binding protein) or fibrinolysis (staphylokinase; SAK). Some of these factors act host-specific (Viana et al., 2010). Moreover, the Sa3int-phage encoded SAK was mostly absent from the murine strains (see above, Table 3). Hence, we tested whether murine *S. aureus* strains are superior to human strains in coagulating murine plasma. In general, murine plasma coagulated more slowly than human plasma. During the first 4 h, however, coagulation of murine plasma was more advanced after incubation with murine than with human CC88 isolates (Figure 3). This may suggest that murine CC88 *S. aureus* isolates have evolved means to specifically modulate the murine coagulation system.

### Colonized laboratory mice mount a systemic immune response to *S. aureus*

We have previously reported that symptom-free human *S. aureus* carriers raise a strong serum IgG response against their colonizing strain (Holtfreter et al., 2006). To test whether this is similar in laboratory mice, we compared antibody profiles of *S. aureus*-free mice with those of symptom-free colonized mice (positive nasopharyngeal culture) and mice with spontaneous PGA. Animals were derived from two Charles River breeding facilities, Kingston and Hollister, and naturally colonized or infected with strains of the CC88 or CC1 lineage, respectively (Table S5). Importantly, colonized mice showed a significant systemic IgG response against extracellular staphylococcal proteins, whereas SOPF mice were immunologically naïve (Figure S2).

**Figure 4:**
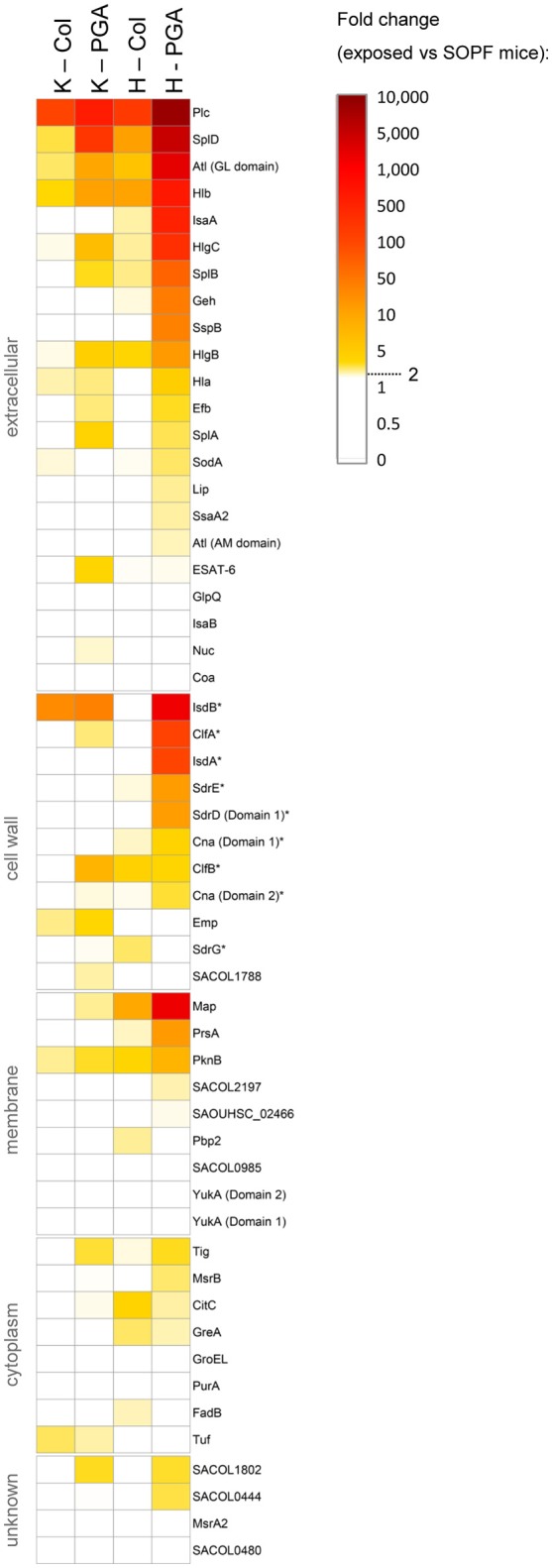
*****S. aureus***-exposed laboratory mice mount a serum IgG response against a range of ***S. aureus*** proteins, including vaccine candidates. (A)** Antigen-specific murine serum IgG was measured using FlexMap® technology. We measured the strain-specific antibody response against 58 recombinant *S. aureus* antigens in colonized symptom-free mice as well as in mice with spontaneous infections (PGA) from Charles River breeding facilities at Kingston and Hollister. SOPF mice served as negative controls. Group size was *n* = 5. Mice from Kingston were exposed to a CC88 isolate, while mice from Hollister were exposed to a CC1 isolate. Fold-changes of exposed mice as compared to the SOPF controls are depicted. Values below 2.0 are displayed in white. Vaccine candidates from previous or ongoing human clinical trials are labelled with an asterix (^*^). Col, colonization; PGA, preputial gland adenitis; K, Kingston (mice were exposed to CC88 isolates); H, Hollister (mice were exposed to CC1 isolates); MFI, Mean fluorescence intensity.

To characterize the induced antibody response in more detail, we quantified serum IgG binding to a panel of 58 recombinant *S. aureus* proteins, including vaccine candidates, using the xMAP® technology. A 2-fold rise in antibody titers in exposed vs. SOPF mice was considered as biologically relevant. A total of seven *S. aureus* proteins were recognized by all *S. aureus*-exposed mice: Plc, Atl, HlgB, HlgC, Hlb, SplD, and PknB (Figure 4, Table S6). Moreover, colonized mice were primed against vaccine candidates from previous or ongoing human clinical trials, i.e., ClfB, Cna, Hla, IsdB, SdrE, and SdrG (Fowler and Proctor, 2014). Notably, colonized mice from both facilities showed more than 100-fold higher Plc-specific antibody titers than SOPF mice, which makes Plc a suitable candidate for a serological screening assay for *S. aureus* exposure (Figure S2).

Antibody titers against numerous antigens were higher in infected mice from Hollister (CC1) than in their counterparts from Kingston (CC88) (Figure 4, Table S6). Since all tested antigens are encoded in the CC88 genome (unpublished data, S. Holtfreter), the more pronounced immune response might be due to differences in the *in vivo* behavior of CC1 and CC88 isolates.

In summary, naturally colonized mice mount a systemic immune response against numerous *S. aureus* antigens, including vaccine candidates from clinical trials.

## Discussion

### SPF mice are frequently colonized with *S. aureus*

Many *S. aureus* researchers doubt that mice are a suitable surrogate host for *S. aureus* research, because mice are not considered to be natural hosts of these bacteria (Cuny et al., 2010; McCarthy and Lindsay, 2010; Capparelli et al., 2011; Mulcahy et al., 2012). In this study, we report that SPF laboratory mice from all major vendors are colonized with *S. aureus* at varying rates (0–20.9%). This is not surprising, because *S. aureus* is considered an opportunistic pathogen in mice and is, therefore, not routinely excluded from SPF barrier rooms. Of note, vendors respond very differently to the presence of opportunistic agents. Some vendors tolerate opportunistic agents in SPF colonies, whereas other vendors test and cull mice to stop the spread. In Europe, publishing *S. aureus* test results of SPF mice is not required according to the FELASA recommendations (Mahler Convenor et al., 2014). In consequence, European researchers must approach each company to obtain information about the *S. aureus* colonization status of their mouse strain of interest. Alternatively, SOPF animals from all vendors are by definition *S. aureus*-free.

### *S. aureus* is readily transmitted from parents to offspring, which in turn becomes persistently colonized

We demonstrated that *S. aureus* are readily transmitted from parents to offspring, which becomes persistently colonized. In line with this, retrospective data on the *S. aureus* prevalence in SPF barriers at Charles River showed that mouse colonies are not able to clear *S. aureus*, once the bacteria have been introduced into a colony (personal communication, K. Pritchett-Corning). These findings imply that *S. aureus* is maintained by efficient vertical transmission, and that early exposure may be decisive for long-term colonization, possibly due to early occupation of niches in the gut and nose. In contrast, human to mouse transmission or acquisition from the environment appears to be much less frequent. In this study, the *S. aureus* strains predominating in animal facilities (CC88 lineage) are rarely found in the human population. Moreover, we observed no concordance of murine and human *S. aureus* isolates at a local university-associated animal facility. This contrasts studies from the 1970s and 80s, which reported similar phage patterns between isolates from care takers and infected mice (Blackmore and Francis, 1970; Needham and Cooper, 1976). This suggests that nowadays a host jump is efficiently limited by using protected cage changing stations and isolation housing (with *S. aureus* regarded as an unwanted pathogen), performing regular microbiological screenings and adhering to a strict hygiene practice (Baker, 2003).

#### CC88 is the predominant lineage among *S. aureus* isolates from laboratory mice

The predominant lineage among the murine isolates was CC88, which is known for causing an outbreak of PGA at the animal facility in Auckland University (Holtfreter et al., 2013). Remarkably, CC88 strains (1) have been persisting in the Charles River facilities for at least a decade, (2) were efficiently transmitted from murine parents to offspring, and (3) were detected in animal facilities around the globe (e.g., New Zealand, US and Germany). This strongly suggest that laboratory mice are a major reservoir for this CC88 (sub-) lineage. We can only speculate on the origin of the murine CC88 lineage. CC88 strains have previously not been associated with animals. Even though CC88 MSSA and MRSA are extremely rare among the North-American and European *S. aureus* populations (Gorwitz et al., 2008; Holtfreter et al., 2016), outbreaks of CC88 CA-MRSA infections have been reported from Asia and Africa (Ghebremedhin et al., 2009; Zhang et al., 2009). Hence, the murine CC88 population in laboratory mice might be the result of a host jump by *S. aureus* strains of human origin, followed by genetic adaptation to mice.

Strains of other lineages, i.e., CC5, CC8, CC15, CC25, CC30, accounted for 37.4% of the murine isolates. These lineages are commonly found in humans, though CC5- and CC8-sublineages are also frequently observed in poultry, and horses, respectively (Lowder et al., 2009; McCarthy et al., 2012). Whether these murine isolates represent a recent human-to-mouse transmission or rather mouse-adapted murine sublineages can only be clarified by whole genome sequencing. Indeed, some of these strains showed features of mouse adaptation, though groups were small. For example, murine CC8 strains lacked the MGE-encoded SAg genes while all human strains were SAg gene-positive. Likewise, ampicillin resistance was rarely observed among murine CC5 and CC8 strains.

We also detected lineages referred to as livestock-associated, i.e., ST72 and ST188 (Sung et al., 2008; McCarthy et al., 2012). Of note, the murine strains were all methicillin-sensitive, suggesting either a transmission of LA-MSSA strains or of LA-MRSA with subsequent loss of the SCCmec element (McCarthy et al., 2012; Cuny et al., 2015). The latter seems unlikely, since LA-MRSA have never been detected in rodents at Charles River facilities (personal communication, K. Pritchett-Corning).

### Adaptation to murine host involves the loss of MGEs encoding human-specific virulence factors

Our comparisons of human and murine *S. aureus* strains suggest that adaptation to the murine host could involve the loss of Sa3int phages, SAg-encoding MGEs and antibiotic resistance genes. Sa3int phages encode immune evasion factors but also destroy the *hlb* gene upon integration into the bacterial chromosome (Figure S1). In the murine system, the IEC-encoded factors show no or negligible activity (Gladysheva et al., 2003; de Haas et al., 2004; Holtfreter and Broker, 2005; Rooijakkers et al., 2005), whereas the sphingomyelinase Hlb mediates hemolysis of ruminant erythrocytes and is an important virulence factor in mouse infection models (Katayama et al., 2013). Thus, in the murine host the disadvantage due to loss of Hlb clearly outweighs the benefits conferred by the IEC-encoded factors. The absence of Sa3int phages, which is accompanied by ß-hemolysis, is indeed a common feature of animal-adapted strains (Markham and Markham, 1966; Sung et al., 2008). Twenty–six percentage of the murine isolates of various lineages were IEC-positive. Whole genome sequencing will reveal whether the murine strains encode allelic, possibly mouse-adapted, variants of the IEC-encoded virulence factors. Alternatively, the presence of IEC-encoding Sa3int phages might indicate a recent human-to-mouse-transmission.

Another molecular correlate of host adaptation was the loss of SAg-encoding MGEs. *S. aureus* SAgs activate human T cells in the picomolar concentration range *in vitro* but show a 10–100-fold reduced activity on murine T cells (Holtfreter and Broker, 2005). Elimination of SAg genes in murine *S. aureus* strains, wherever their genetic location permits this, underlines the inefficiency of SAg toxins in mice. Finally, penicillin resistance, which is mediated by the production of the *blaZ*-encoded ß-lactamase, was rare among murine isolates. Maybe the host jump occurred before the spread of ampicillin resistance in the *S. aureus* population (i.e., 1950s; Chambers and Deleo, 2009). Alternatively, the ß-lactamase-encoding plasmids or transposons might have been lost following the host jump (Lyon and Skurray, 1987). Commercial vendors do not use antibiotics in barriers, thereby removing the selective pressure for maintaining the resistance gene.

The ability to coagulate plasma or blood, respectively, is an essential virulence trait of *S. aureus*, and some pro- or anti-coagulatory factors are host-specific (Viana et al., 2010). This makes it a strong and easily addressable indicator for host-adaptation. Here, we report that murine CC88 isolates showed a tendency to coagulate murine plasma faster than matched human CC88 isolates. Further experiments are necessary to confirm that there is a biologically relevant difference in the coagulation rate between human and murine *S. aureus* isolates. Viana *et al*. reported that ruminant strains, but not human isolates, had the capacity to stimulate clotting of ruminant plasma (Viana et al., 2010). In some ruminant strains this effect is based on a MGE-encoded paralogue of the van Willebrandt factor binding protein.

### Colonized laboratory mice mount a systemic immune response against *S. aureus*, which could bias infection experiments

The frequent colonization of laboratory mice with *S. aureus* is of concern, because it could significantly affect *S. aureus* infection and vaccination studies. Here, we show that colonized SPF mice mount a systemic antibody response against a panel of *S. aureus* proteins, including numerous *S. aureus* vaccine candidates from past or current clinical trials, i.e., Hla, ClfA, IsdA, IsdB, SdrD, SdrE, SdrG, Cna, and ClfB (Fowler and Proctor, 2014). In contrast, commensal gut bacteria usually do not elicit a systemic IgG response but a local IgA response. This suggests that *S. aureus* is more aggressive than gut commensals and probably induces minor subclinical infections in mice which then trigger a systemic immune response as it has been suggested for humans as well (Broker et al., 2014).

Importantly, immune priming of laboratory mice prior to experimental infection or vaccination may strongly influence the outcome (Broker et al., 2014; Murphy et al., 2014; Brown et al., 2015; Stentzel et al., 2015). Unrecognized colonization with *S. aureus* may, therefore, be a significant confounder in experimental studies of infection and vaccination of mice, especially if prior exposure to *S. aureus* is variable. We are convinced that *S. aureus* research and vaccine development will benefit from well-defined mouse cohorts and encourage researchers to ensure that they work with either *S. aureus*-free or consistently -primed mice.

Since *S. aureus* screening results from commercial vendors are not available for SPF animals from European facilities, a microbiological or serological screening assay will be of advantage. Nose homogenates or stool samples are suitable for microbiological screenings, but the available selective media are error prone (unpublished data, S. Holtfreter). In contrast, Plc, for which we observed a more than 100-fold increase in antibody titers in *S. aureus*-exposed mice, could be a robust and sensitive marker for a serological screening assay for *S. aureus* exposure.

Since mice are natural hosts of *S. aureus*, they could be better surrogate models for infection and vaccination studies than previously assumed. Robust and clinically relevant infection models are mandatory for the development of new strategies to prevent or treat *S. aureus* infection. Our data demonstrate that laboratory mice are better models for colonization and infection studies than previously assumed. Just like humans, laboratory mice are persistently colonized with *S. aureus*, mount a systemic immune response upon colonization, and frequently suffer from abscess formation. Moreover, the use of mouse-adapted *S. aureus* strains in their natural host—the mouse—promises to provide a more physiological model for studying *S. aureus* host interaction and testing novel therapeutics. Such strains can show a better fitness and virulence in mouse colonization and infection models, respectively (Holtfreter et al., 2013). However, one has to keep in mind that mice and mouse-adapted strains are not suited to study the effect of human-specific virulence factors, such as PVL and SAgs (Loffler et al., 2010).

In summary, this study clearly shows that laboratory mice are natural hosts of *S. aureus* with CC88 being the dominant murine *S. aureus* lineage. *S. aureus* spreads in mouse colonies by transmission from parents to offspring rather than via contaminated caretakers and can persistently colonize young mice. The bacteria have adapted to their murine host environment by various means including removal of superfluous genetic material (Sa3int phage, SAg-encoding MGEs). Most importantly, *S. aureus* colonization primes the adaptive immune system, which is of concern because it will influence experimental infection and vaccination studies. These findings underline the importance of using *S. aureus*-free or consistently primed mice for infection experiments since prior colonization could severely bias disease outcome and also affect the reproducibility of *in vivo* infection experiments.

## Author contributions

SH, SW, FS, KP, and DS designed experiments, SH wrote the manuscript. DS, DG, PT, KP, SJ, KR, JG, NS, SMi, SB, Jv, RF, SMo, and BU performed the experiments. All authors analyzed and interpreted data. SH, SW, FS, SB, DS, BB, KP, and SM participated in the interpretation and discussion of the results. All authors read and approved the final manuscript.

## Funding

This work was supported by a Sir Charles Hercus Fellowship from the Health Research Council of New Zealand (SW, 09/099, http://www.hrc.govt.nz/), Deutsche Forschungsgemeinschaft (SH, GRK1870, http://www.dfg.de/) and the Bundesministerium für Bildung und Forschung (BB, InVAC, https://www.bmbf.de/). The funders had no role in study design, data collection and interpretation, or the decision to submit the work for publication.

### Conflict of interest statement

SMo is an employee of Alere Technologies, a company that manufactures microarrays. The company had no influence on the study design, experiments, data interpretation, and publication. KP and RF previously worked with Charles River, USA. The company had no influence on the study design, experiments, data interpretation, and publication. The other authors declare that the research was conducted in the absence of any commercial or financial relationships that could be construed as a potential conflict of interest.
