# Supplementary material for: Laboratory Mice Are Frequently Colonized with Staphylococcus aureus and Mount a Systemic Immune Response—Note of Caution for In vivo Infection Experiments
Source: Front Cell Infect Microbiol. 2017 May 2;7:152. doi: 10.3389/fcimb.2017.00152 (PMC5411432; doi:10.3389/fcimb.2017.00152)
Supplement: Supplementary file 1 [file Table1.PDF]

S1 Table: Time distance between recycling an SPF barrier room and the first occurence of *S. aureus* within this area.

SPF Barrier	Location	Recycling date	First time positive for *S. aureus*
K95	Kingston, US	Oct-97	Mar-02
K96	Kingston, US	Jun-01	Apr-02
R02	Raleigh, US	Jan-06	Feb-07
R03	Raleigh, US	Oct-07	Jun-09
K94	Kingston, US	Feb-05	negative
