# Supplementary material for: Laboratory Mice Are Frequently Colonized with Staphylococcus aureus and Mount a Systemic Immune Response—Note of Caution for In vivo Infection Experiments
Source: Front Cell Infect Microbiol. 2017 May 2;7:152. doi: 10.3389/fcimb.2017.00152 (PMC5411432; doi:10.3389/fcimb.2017.00152)
Supplement: Supplementary file 2 [file Table2.PDF]

S2 Table: Genotype, virulence genes, phage patterns and ampicillin resistance of colonizing *S. aureus* isolates from healthy blood donors.

		spa genotyping				Virulence genes							Phage genes												
Strain ID	Year	*spa* type	*spa* repeats	MLST	deduced MLST CC[1]	MGE-encoded SAgs	*egc* SAgs	*agr*	*eta*	*etd*	*pvl*	*mecA*	*Sa1int*	*Sa2int*	*Sa3int*	*Sa4int*	*Sa5int*	*Sa6int*	*Sa7int*	*sea*	*sep*	*sak*	*chp*	*scn*	AmpR
SH044-1	2005	t127	07-23-21-16-34-33-13	ND	CC1	a h k q	-	3	-	-	-	-	-	+	+	-	-	-	-	+	-	+	-	+	+
SH097-2	2005	t1491	07-23-21-17-13-34-34-16-34-33-13	ND	CC1	h	-	3	-	-	-	-	-	-	-	-	-	-	-	-	-	-	-	-	-
SH033-1	2005	t002	26-23-17-34-17-20-17-12-17-16	ND	CC5	b k q d j r	g i m n o	2	-	-	-	-	-	-	+	-	-	-	-	-	-	+	+	+	+
SH042-1	2005	t002	26-23-17-34-17-20-17-12-17-16	ND	CC5	p	g i m n o	2	-	-	-	-	-	-	+	-	-	-	-	-	+	+	+	+	+
SH076-1	2005	t002	26-23-17-34-17-20-17-12-17-16	ND	CC5	-	g i m n o	2	-	-	-	-	-	-	+	-	-	-	-	-	-	+	-	+	+
SH093-1	2005	t002	26-23-17-34-17-20-17-12-17-16	ND	CC5	-	g i m n o	2	-	-	-	-	-	-	-	-	-	-	-	-	-	-	-	-	+
SH137-2	2005	t002	26-23-17-34-17-20-17-12-17-16	ND	CC5	p	g i m n o	2	-	-	-	-	-	-	+	-	+	-	-	-	+	+	+	+	+
T009-2	2002	t002	26-23-17-34-17-20-17-12-17-16	ND	CC5	p	g i m n o	2	-	-	-	-	-	-	+	-	-	-	-	-	+	+	+	+	+
T161-1	2002	t002	26-23-17-34-17-20-17-12-17-16	ND	CC5	-	g i m n o	2	-	-	-	-	-	-	+	-	-	-	-	-	-	+	-	+	-
SH081-2	2005	t091	07-23-21-17-34-12-23-02-12-23	ND	CC7	p	-	1	-	-	-	-	+	-	+	-	-	-	-	-	+	+	-	+	+
T002-2	2002	t091	07-23-21-17-34-12-23-02-12-23	ND	CC7	p	-	1	-	-	-	-	+	-	+	-	-	-	-	-	+	+	-	+	+
T009-1	2002	t091	07-23-21-17-34-12-23-02-12-23	ND	CC7	p	-	1	-	-	-	-	-	-	+	-	-	-	-	-	+	+	-	+	+
T157-1	2002	t091	07-23-21-17-34-12-23-02-12-23	ND	CC7	p	-	1	-	-	-	-	-	-	+	-	-	-	-	-	+	+	-	+	+
SH010-1	2005	t008	11-19-12-21-17-34-24-34-22-25	ND	CC8	d j r	-	1	-	-	-	-	-	-	+	-	-	-	-	-	-	+	-	+	-
SH016-1	2005	t008	11-19-12-21-17-34-24-34-22-25	ND	CC8	d j r	-	1	-	-	-	-	-	-	-	-	-	-	-	-	-	-	-	-	+
SH051-2	2005	t008	11-19-12-21-17-34-24-34-22-25	ND	CC8	d j r	-	1	-	-	-	-	-	-	+	-	-	-	-	-	-	+	-	+	+
SH100-1	2005	t008	11-19-12-21-17-34-24-34-22-25	ND	CC8	c l	-	1	-	-	-	-	-	-	-	-	-	-	-	-	-	-	-	-	+
SH115-2	2005	t008	11-19-12-21-17-34-24-34-22-25	ND	CC8	d j r	-	1	-	-	-	-	-	-	+	-	-	-	-	-	-	+	-	+	+
T041-1	2002	t008	11-19-12-21-17-34-24-34-22-25	ND	CC8	d j r	-	1	-	-	-	-	-	-	+	-	-	-	-	-	-	+	-	+	+
T051-2	2002	t008	11-19-12-21-17-34-24-34-22-25	ND	CC8	d j r	-	1	-	-	-	-	-	-	+	-	-	-	-	-	-	+	-	+	+
T145-1	2002	t711	04-21-17-34-24-34-22-25	ND	CC8	b k q	-	1	-	-	-	-	-	+	+	-	-	-	-	-	-	+	-	+	-
T169-1	2002	t711	04-21-17-34-24-34-22-25	ND	CC8	b k q d j r	-	1	-	-	-	-	-	+	+	-	-	-	-	-	-	+	-	+	+
T198-1	2002	t711	04-21-17-34-24-34-22-25	ND	CC8	b q d j r	-	1	-	-	-	-	-	+	+	-	-	-	-	-	-	+	-	+	+
SH131-1	2005	t723	11-19-12-34-22-25	ND	CC8	a d j r	-	1	-	-	-	-	-	-	+	-	-	-	-	+	-	+	-	+	+
SH059-1	2005	t156	07-23-12-33-22-17	ND	CC12	c l p	-	2	-	-	-	-	-	-	+	-	-	-	-	-	+	+	-	+	-
T194-1	2002	t156	07-23-12-33-22-17	ND	CC12	c l p	-	2	-	-	-	-	-	-	+	-	-	-	-	-	+	+	-	+	-
SH027-1	2005	t084	07-23-12-34-34-12-12-23-02-12-23	ND	CC15	-	-	2	-	-	-	-	-	-	-	-	-	-	-	-	-	-	+	+	+
SH028-1	2005	t084	07-23-12-34-34-12-12-23-02-12-23	ND	CC15	-	-	2	-	-	-	-	-	-	-	-	-	-	-	-	-	-	+	+	+
SH070-1	2005	t084	07-23-12-34-34-12-12-23-02-12-23	ND	CC15	-	-	2	-	-	-	-	-	-	-	-	-	-	-	-	-	-	+	+	+
SH130-1	2005	t084	07-23-12-34-34-12-12-23-02-12-23	ND	CC15	-	-	2	-	-	-	-	-	-	-	-	-	+	-	-	-	-	+	+	-
T093-1	2002	t084	07-23-12-34-34-12-12-23-02-12-23	ND	CC15	-	-	2	-	-	-	-	+	-	-	-	-	+	-	-	-	-	+	+	+
T099-1	2002	t084	07-23-12-34-34-12-12-23-02-12-23	ND	CC15	-	-	2	-	-	-	-	-	+	-	-	-	-	-	-	-	-	+	+	+
SH126-2	2005	t085	07-23-12-34-34-12-23-02-12-23	ND	CC15	-	-	2	-	-	-	-	-	+	-	-	-	-	-	-	-	-	+	+	-
SH019-2	2005	t120	07-23-12-12-34-34-12-12-23-02-12-23	ND	CC15	-	-	2	-	-	-	-	-	+	-	-	-	-	-	-	-	-	+	+	+
T202-2	2002	t346	07-23-12-34-12-12-23-02-12-23	ND	CC15	-	-	2	-	-	-	-	-	+	-	-	-	-	-	-	-	-	+	+	+
SH072-1	2005	t005	26-23-13-23-31-05-17-25-17-25-16-28	ND	CC22	-	g i m n o	1	-	-	-	-	-	-	+	-	-	-	-	-	-	+	+	+	+
SH073-1	2005	t005	26-23-13-23-31-05-17-25-17-25-16-28	ND	CC22	-	g i m n o	1	-	-	-	-	-	-	+	-	-	-	-	-	-	+	+	+	+
SH055-1	2005	t1648	26-23-29-17-25-17-25-28	ND	CC22	-	g i m n o	1	-	-	-	-	-	-	+	-	-	-	-	-	-	+	+	+	+
SH024-2	2005	t541	26-17-25-17-25-16-28	ND	CC22	-	g i m n o	1	-	-	-	-	-	-	+	-	-	-	-	-	-	+	+	+	-
T162-1	2002	t078	04-21-12-41-20-17-12-12-17	ND	CC25	b	g i m n o	1	-	-	-	-	-	+	+	-	-	-	-	-	-	+	+	+	+
T171-1	2002	t078	04-21-12-41-20-17-12-12-17	ND	CC25	b	g i m n o	1	-	-	-	-	-	+	+	-	-	-	-	-	-	+	+	+	+
T192-1	2002	t078	04-21-12-41-20-17-12-12-17	ND	CC25	-	g i m n o	1	-	-	-	-	-	+	+	-	-	-	-	-	-	+	+	+	-
T097-1	2002	t258	04-21-12-41-20-17-12-12-12-17	ND	CC25	-	g i m n o	1	-	-	-	-	-	+	+	-	-	-	-	-	-	+	+	+	+
SH039-1	2005	t012	15-12-16-02-16-02-25-17-24-24	ND	CC30	tst	g i m n o u	3	-	-	-	-	-	+	+	-	-	-	-	-	-	+	+	+	+
SH077-1	2005	t012	15-12-16-02-16-02-25-17-24-24	ND	CC30	-	g i m n o u	3	-	-	-	-	-	-	+	-	-	-	-	-	-	+	-	+	+
SH112-2	2005	t012	15-12-16-02-16-02-25-17-24-24	ND	CC30	a tst	g i m n o u	3	-	-	-	-	-	+	+	-	-	-	-	+	-	+	+	+	+
T043-1	2002	t012	15-12-16-02-16-02-25-17-24-24	ND	CC30	a tst	g i m n o u	3	-	-	-	-	-	+	+	-	-	-	-	+	-	+	+	+	-
T098-1	2002	t012	15-12-16-02-16-02-25-17-24-24	ND	CC30	a	g i m n o u	3	-	-	-	-	-	+	+	-	+	-	-	+	-	+	+	+	+
T098-2	2002	t012	15-12-16-02-16-02-25-17-24-24	ND	CC30	-	g i m n o u	3	-	-	-	-	-	+	-	-	+	-	-	-	-	-	-	-	+
T100-1	2002	t012	15-12-16-02-16-02-25-17-24-24	ND	CC30	tst	g i m n o u	3	-	-	-	-	-	+	+	-	-	-	-	-	-	+	+	+	+
T124-1	2002	t012	15-12-16-02-16-02-25-17-24-24	ND	CC30	tst	g i m n o u	3	-	-	-	-	-	-	+	-	+	-	-	-	-	+	+	+	+
T141-1	2002	t012	15-12-16-02-16-02-25-17-24-24	ND	CC30	a tst	g i m n o u	3	-	-	-	-	-	+	+	-	+	-	-	+	-	+	+	+	+
T190-2	2002	t012	15-12-16-02-16-02-25-17-24-24	ND	CC30	tst	g i m n o u	3	-	-	-	-	-	+	-	-	+	-	-	-	-	-	-	-	-
T135-1	2002	t017	15-12-16-16-02-16-02-25-17-24-24	ND	CC30	a	g i m n o u	3	-	-	-	-	-	+	+	-	-	-	-	+	-	+	+	+	-
SH105-1	2005	t018	15-12-16-02-16-02-25-17-24-24-24	ND	CC30	tst	g i m n o u	3	-	-	-	-	-	-	+	-	+	-	-	-	-	+	+	+	+
SH032-1	2005	t019	08-16-02-16-02-25-17-24	ND	CC30	a tst	g i m n o u	3	-	-	-	-	-	+	+	-	-	+	-	+	-	+	+	+	+
SH109-1	2005	t021	15-12-16-02-16-02-25-17-24	ND	CC30	-	g i m n o u	3	-	-	-	-	-	+	+	-	+	-	-	-	-	-	+	+	-
SH067-1	2005	t122	08-16-02-16-02-25-17-24-24	ND	CC30	a tst	g i m n o u	3	-	-	-	-	-	-	+	-	+	-	-	+	-	+	+	+	+
SH079-1	2005	t122	08-16-02-16-02-25-17-24-24	ND	CC30	a tst	g i m n o u	3	-	-	-	-	-	+	+	-	+	-	-	+	-	+	+	+	+
T039-2	2002	t122	08-16-02-16-02-25-17-24-24	ND	CC30	tst	g i m n o u	3	-	-	-	-	-	+	+	-	-	-	-	-	-	+	-	+	+
SH064-1	2005	t1504	15-12-16-16-02-25-17-24	ND	CC30	a tst	g i m n o u	3	-	-	-	-	-	+	+	-	-	-	-	+	-	+	+	+	+
SH065-2	2005	t1504	15-12-16-16-02-25-17-24	ND	CC30	a tst	g i m n o u	3	-	-	-	-	-	+	+	-	-	-	-	+	-	+	+	+	+
SH069-1	2005	t1641	15-12-16-02-16-02-25-16-17-24	ND	CC30	tst	g i m n o u	3	-	-	-	-	-	-	-	-	-	-	-	-	-	-	-	-	+
T061-1	2002	t1642	15-16-02-25-17-24-24	ND	CC30	a	g i m n o u	3	-	-	-	-	-	+	+	-	-	-	-	+	-	+	+	+	+
SH020-1	2005	t1643	04-44-54-31-12-16-34-16-12-33-34	ND	CC30	h tst	g i m n o u	3	-	-	-	-	-	-	+	-	-	-	-	-	-	+	+	+	+
SH106-2	2005	t1650	15-12-16-16-02-16-02-25-17-25	ND	CC30	-	g i m n o u	3	-	-	-	-	-	+	+	-	-	-	-	-	-	+	+	+	+
T172-1	2002	t1660	15-12-16-16-16-16-16-02-16-02-25-17-24	ND	CC30	-	g i m n o u	3	-	-	-	-	-	-	-	-	-	-	-	-	-	-	-	-	-
T077-1	2002	t1672	15-12-16-02-16-16-02-16-02-25-17-24-24	ND	CC30	a tst	g i m n o u	3	-	-	-	-	-	-	+	-	-	-	-	+	-	+	+	+	+
SH046-1	2005	t275	15-12-16-02-25-17-24-24	ND	CC30	tst	g i m n o u	3	-	-	-	-	-	+	+	-	+	-	-	-	-	+	+	+	-
T169-2	2002	t318	15-12-16-16-02-16-02-25-17-24	ND	CC30	-	g i m n o u	3	-	-	-	-	-	-	-	-	-	+	-	-	-	-	-	-	-
T035-1	2002	t342	15-12-16-02-16-02-25-17	ND	CC30	-	g i m n o u	3	-	-	-	-	-	-	+	-	+	-	-	-	-	+	+	+	+
T037-1	2002	t840	15-12-02-16-02-25-17-24-24	ND	CC30	tst	g i m n o u	3	-	-	-	-	-	-	+	-	+	-	-	-	-	+	+	+	+
SH121-1	2005	t964	08-16-16-02-16-02-25-17-24	ND	CC30	-	g i m n o u	3	-	-	-	-	-	-	+	-	-	+	-	-	-	+	+	+	-
T200-1	2002	t1664	04-33-31-12-16-16-12-33-34	ND	CC34	a h tst	g i m n o u	3	-	-	-	-	-	+	+	-	+	-	-	+	-	+	+	+	+
T020-1	2002	t1670	04-44-33-31-12-16-34-12-33-34	ND	CC34	b h l tst	g i m n o u	3	-	-	-	-	-	-	+	-	+	+	-	-	-	+	-	+	+
T184-2	2002	t004	09-02-16-13-13-17-34-16-34	ND	CC45	-	g i m n o	1	-	-	-	+	-	-	+	-	+	-	-	-	-	+	+	+	+
SH001-2	2005	t015	08-16-02-16-34-13-17-34-16-34	ND	CC45	c l	g i m n o	1	-	-	-	-	-	-	-	-	-	-	-	-	-	+	+	+	+
T052-1	2002	t015	08-16-02-16-34-13-17-34-16-34	ND	CC45	a c l	g i m n o	1	-	-	-	-	-	-	+	-	-	-	-	+	-	+	+	+	-
T166-2	2002	t015	08-16-02-16-34-13-17-34-16-34	ND	CC45	c l	g i m n o	1	-	-	-	-	-	-	+	-	-	-	-	-	-	+	+	+	+
T108-1	2002	t050	08-16-02-16-34-34-17-34-16-34	ND	CC45	c l	g i m n o	1	-	-	-	-	-	-	-	-	+	-	-	-	-	+	+	+	-
T085-1	2002	t065	09-02-16-34-13-17-34-16-34	ND	CC45	-	g i m n o	1	-	-	-	-	+	-	+	-	+	+	-	-	-	+	+	+	+
SH024-1	2005	t1238	08-104-02-16-34-13-17-34-16-34	ND	CC45	c l	g i m n o	1	-	-	-	-	-	-	+	-	-	-	-	-	-	+	+	+	-
SH056-1	2005	t1647	08-13-16-34-16-34	ST45	CC45	c l	g i m n o	1	-	-	-	-	+	-	+	-	-	-	-	-	-	+	+	+	-
T088-2	2002	t1666	08-16-02-83-34-13-17-34-83-34	ND	CC45	c l	g i m n o	1	-	-	-	-	-	-	+	-	-	-	-	-	-	+	+	+	+
SH013-2	2005	t302	08-16-02-16-34-13-17-13-16-34	ND	CC45	-	g i m n o	1	-	-	-	-	-	-	-	-	-	-	-	-	-	+	+	+	-
T179-1	2002	t302	08-16-02-16-34-13-17-13-16-34	ND	CC45	c l	g i m n o	1	-	-	-	-	-	-	+	-	+	-	-	-	-	+	+	+	+
SH018-1	2005	t246	04-17-23-24-20-17-25	ND	CC50	-	i m n o u	4	-	-	-	-	-	-	+	-	-	-	-	-	-	+	+	+	-
SH015-1	2005	t056	04-20-12-17-20-17-12-17-17	ND	CC101	-	-	1	-	-	-	-	-	-	+	-	-	-	-	-	-	+	-	+	-
SH021-2	2005	t056	04-20-12-17-20-17-12-17-17	ND	CC101	-	-	1	-	-	-	-	-	-	+	-	-	-	-	-	-	+	-	+	-
SH022-1	2005	t056	04-20-12-17-20-17-12-17-17	ND	CC101	-	-	1	-	-	-	-	-	-	+	-	-	-	-	-	-	+	-	+	-
SH133-1	2005	t056	04-20-12-17-20-17-12-17-17	ND	CC101	-	-	1	-	-	-	-	-	+	-	-	-	-	-	-	-	-	-	-	-
SH145-1	2005	t056	04-20-12-17-20-17-12-17-17	ND	CC101	-	-	1	-	-	-	-	-	-	+	-	-	-	-	-	-	+	-	+	-
T011-1	2002	t056	04-20-12-17-20-17-12-17-17	ND	CC101	-	-	1	-	-	-	-	-	+	+	-	-	-	-	-	-	+	-	+	-
T029-2	2002	t056	04-20-12-17-20-17-12-17-17	ND	CC101	-	-	1	-	-	-	-	-	-	+	-	-	-	-	-	-	+	-	+	-
T055-1	2002	t056	04-20-12-17-20-17-12-17-17	ND	CC101	-	-	1	-	-	-	-	-	-	+	-	-	-	-	-	-	+	-	+	-
T105-2	2002	t056	04-20-12-17-20-17-12-17-17	ND	CC101	-	-	1	-	-	-	-	-	+	+	-	-	-	-	-	-	+	-	+	-
T201-1	2002	t056	04-20-12-17-20-17-12-17-17	ND	CC101	-	-	1	-	-	-	-	-	-	+	-	-	-	-	-	-	+	-	+	-
T142-2	2002	t493	04-34-17-66-32-17-23-24	ND	ST182	h	g i m n o u	1	-	-	-	-	-	-	+	-	+	-	-	-	-	+	+	+	-
T148-2	2002	t1362	08-12-16-34-02-43-34-16-02-17-16	ND	CC395	a c l tst	-	1	-	-	-	-	+	-	+	-	-	-	-	+	-	-	+	+	-
T191-1	2002	t1645	08-12-16-02-43-34-16-16-02-17-16	ND	CC395	a c l tst	-	1	-	-	-	-	+	-	+	-	-	-	-	+	-	+	+	+	+
T110-1	2002	t1651	08-16-02-43-34-16-02-17-16	ND	CC395	a k q c l tst	-	1	-	-	-	-	+	-	-	-	-	-	-	+	-	+	+	+	-
T166-1	2002	t1655	08-16-02-43-34-16-16-02-17-16	ST395	CC395	k q	-	1	-	-	-	-	-	-	+	-	-	-	-	-	-	+	+	+	+
T132-1	2002	t1669	08-16-34-02-43-13-16-16-02-17-16	ST395	CC395	a	-	1	-	-	-	-	+	-	+	-	-	-	-	+	-	+	+	+	+
SH017-2	2005	t1209	15-12-16	ND	excluded	a	g i m n o u	3	-	-	-	-	-	+	+	-	+	-	-	+	-	+	+	+	+
SH048-1	2005	t026	08-16-34	ND	excluded	c l	g i m n o	1	-	-	-	-	-	-	+	-	-	-	-	-	-	+	+	+	+
T118-1	2002	t118	11-25	ND	excluded	d j r	-	1	-	-	-	-	-	-	+	-	-	-	-	-	-	+	-	+	+
T054-1	2002	t1680	121-17-17-17-17	ND	singleton	b	-	4	-	-	-	-	-	-	-	-	-	-	-	-	-	-	-	-	-

[1] *spa* types were clustered by BURP analysis into CCs and corresponding MLST CCs were deduced using the Ridom database.

Key: col, nasal colonization (nasopharyngeal sample); SSTI, skin and soft tissue infection; CRSwNP, chronic rhinosinusitis with nasal polyps; *agr,* accessory gene regulator; Staphylococcal enterotoxins (SEs) are indicated by single letters (a = *sea* , etc.). *Tst,* toxic shock syndrome toxin 1 gene; *egc,* superantigen genes of the enterotoxin gene cluster, i.e. *seg, sei, sem, sen, seo,* and *seu; eta/etd,* exfoliative toxins a and d; *luk-PV,* Panton-Valentine leukocidine gene; *Sa1int, S. aureus* integrase type 1; *sak,* Staphylokinase gene; *chp* , gene encoding the chemotaxis inhibitory protein; *scn,* staphylococcal complement inhibitor gene; AmpR, ampicillin resistance
