# Supplementary material for: Laboratory Mice Are Frequently Colonized with Staphylococcus aureus and Mount a Systemic Immune Response—Note of Caution for In vivo Infection Experiments
Source: Front Cell Infect Microbiol. 2017 May 2;7:152. doi: 10.3389/fcimb.2017.00152 (PMC5411432; doi:10.3389/fcimb.2017.00152)
Supplement: Supplementary file 3 [file Table3.PDF]

S3 Table: Genotype, virulence genes and phage patterns of colonizing *S. aureus* isolates from randomly sampled mice and their animal care takers at a university breeding facility.

					***spa* genotyping**			**Virulence genes**							**Phage genes**									
**Strain ID**	**Isolation year**	**Host species**	**Strain**	**Sampling side**	***spa* type**	***spa* repeats**	**deduced MLST CC[1]**	**MGE-encoded SAgs**	***egc* SAgs**	***agr***	***eta***	***etd***	***pvl***	***mecA***	***Sa1int***	***Sa2int***	***Sa3int***	***Sa4int***	***Sa5int***	***Sa6int***	***Sa7int***	***sak***	***chp***	***scn***
ZSFV 26-4	2013	mouse	C57BL6/N	feces	t127	07-23-21-16-34-33-13	CC1	*h*	-	*3*	-	-	-	-	-	+	-	-	-	-	-	-	-	-
ZSFV 29-2	2013	mouse	C57BL6/N	feces	t127	07-23-21-16-34-33-13	CC1	*h*	-	*3*	-	-	-	-	-	+	-	-	-	-	-	-	-	-
ZSFV 28-2	2013	mouse	C57BL6/N	feces	t6811	07-23-21-16-34-33	CC1	*h*	-	*3*	-	-	-	-	-	+	-	-	-	-	-	-	-	-
ZSFV 35-1	2013	mouse	C57BL6/N albino	feces	t084	07-23-12-34-34-12-12-23-02-12-23	CC15	-	-	*2*	-	-	-	-	-	-	-	-	+	-	-	-	+	+
ZSFV 36-1	2013	mouse	C57BL6/N albino	feces	t084	07-23-12-34-34-12-12-23-02-12-23	CC15	-	-	*2*	-	-	-	-	-	-	-	-	+	-	-	-	+	+
ZSFV 20-2	2013	mouse	C57BL6/N albino	feces	t491	26-23-12-34-34-12-12-23-02-12-23	CC15	-	-	*2*	-	-	-	-	-	-	-	-	+	-	-	-	+	+
ZSFV 18-3	2013	mouse	C57BL6/N albino	feces	t084	07-23-12-34-34-12-12-23-02-12-23	CC15	-	-	*2*	-	-	-	-	-	-	-	-	+	-	-	-	+	+
ZSFV 14-2	2013	mouse	C57BL6/J	feces	t2311	07-12-12-21-17-13-13-34-34-33-34	CC88	-	-	*3*	-	-	-	-	+	+	+	-	-	-	-	+	+	+
ZSFV 13-2	2013	mouse	C57BL6/J	feces	t6728	07-12-12-21-17-13-34-34-33-34	CC88	-	-	*3*	-	-	-	-	-	+	-	-	-	-	-	-	-	-
ZSFV 17-1	2013	mouse	C57BL6/J	feces	t6728	07-12-12-21-17-13-34-34-33-34	CC88	-	-	*3*	-	-	-	-	-	+	-	-	-	-	-	-	-	-
ZSFV_A	2013	human	N.A.	nose	t902	07-23-31-05-17-25-17-25-16-28	CC22	-	*gimno*	*1*	-	-	-	-	-	-	+	-	-	-	-	+	+	+
ZSFV_B	2014	human	N.A.	nose	t1509	07-23-12-23	CC15	*p*	-	*1*	-	-	-	-	-	-	+	-	-	-	-	+	-	+
ZSFV_C	2013	human	N.A.	nose	t1509	07-23-12-23	CC15	*p*	-	*1*	-	-	-	-	-	-	+	-	-	-	-	+	-	+
ZSFV_D	2014	human	N.A.	nose	t026	08-16-34	CC45	*cl*	*gimno*	*1*	-	-	-	-	+	+	+	-	-	-	-	+	+	+
ZSFV_E	2014	human	N.A.	nose	t2614	08-34-16-34	CC45	*cl*	*gimno*	*1*	-	-	-	-	-	-	+	-	+	-	-	+	+	+

1 *spa* types were clustered by BURP analysis into CCs and corresponding MLST CCs were deduced using the Ridom database.

Key: MGE, mobile genetic element; *agr,* accessory gene regulator; Staphylococcal enterotoxins (SEs) are indicated by single letters (a = *sea* , etc.). *tst,* toxic shock syndrome toxin 1 gene; *egc,* superantigen genes of the enterotoxin gene cluster; i.e. *seg, sei, sem, sen, seo,* and *seu* ; *eta/etd, ex* foliative toxins a and d; *pvl,* Panton-Valentine leukocidine gene; *Sa1int,S. aureus* integrase type 1; *sak,* Staphylokinase gene; *chp,* gene encoding the chemotaxis inhibitory protein; *scn,* staphylococcal complement inhibitor gene
