# Supplementary material for: Laboratory Mice Are Frequently Colonized with Staphylococcus aureus and Mount a Systemic Immune Response—Note of Caution for In vivo Infection Experiments
Source: Front Cell Infect Microbiol. 2017 May 2;7:152. doi: 10.3389/fcimb.2017.00152 (PMC5411432; doi:10.3389/fcimb.2017.00152)
Supplement: Supplementary file 4 [file Table4.PDF]

S4 Table: Genotype, virulence genes, phage patterns and ampicillin resistance of human CC88 *S. aureus* isolates.

				spa genotyping				Virulence genes							Phage genes												
Strain ID	Year	Type of infection	Country	*spa* type	*spa* repeats	MLST	deduced MLST CC[1]	MGE-encoded SAgs	*egc* SAgs	*agr*	*eta*	*etd*	*pvl*	*mecA*	*Sa1int*	*Sa2int*	*Sa3int*	*Sa4int*	*Sa5int*	*Sa6int*	*Sa7int*	*sea*	*sep*	*sak*	*chp*	*scn*	AmpR
F25	2008	SSTI	New Zealand	t11192	07-21-17-34-34-34-34-24-33-34	ST88	CC88	-	-	*3*	-	-	-	-	-	-	+	-	-	-	+	-	-	+	-	+	(+)
sh331195	2008	col	Germany	t1322	07-21-17-34-34-34-33-34	ST88	CC88	*c l tst*	-	*3*	-	-	-	-	-	-	+	-	-	-	-	-	-	+	+	+	+
Z1353	2006	unknown	Australia	t14389	07-12-21-17-13-13-147-34-33-34	ND	CC88	-	-	*3*	-	-	-	+	-	-	+	-	-	-	-	-	-	+	-	+	+
SM_A104	2008	SSTI	Gemany	t1598	07-12-21-13-13-34-34-33-34	ND	CC88	*b*	-	*3*	-	-	+	-	-	+	+	-	-	-	-	-	-	+	-	+	(+)
A50	2007	febrile neutropenia	New Zealand	t186	07-12-21-17-13-13-34-34-33-34	ST78	CC88	-	-	*3*	-	-	-	-	-	+	+	-	-	-	-	-	-	+	-	+	+
M25	2007	endocarditis	New Zealand	t186	07-12-21-17-13-13-34-34-33-34	ST88	CC88	-	-	*3*	+	-	-	-	+	-	+	-	-	-	-	-	-	+	-	+	+
M3	2007	SSTI	New Zealand	t186	07-12-21-17-13-13-34-34-33-34	ST88	CC88	-	-	*3*	-	-	-	-	+	-	+	-	-	-	-	-	-	+	-	+	+
Z1350	2006	unknown	Australia	t186	07-12-21-17-13-13-34-34-33-34	ND	CC88	-	-	*3*	-	-	-	+	-	-	+	-	-	-	-	-	-	+	-	+	+
Z1377	2009	unknown	Australia	t186	07-12-21-17-13-13-34-34-33-34	ND	CC88	*cl*	-	*3*	-	-	-	+	-	-	+	-	-	-	-	-	-	+	-	+	+
sh46713	2012	col	Germany	t2393	07-12-21-17-13-13-13-34-33-34	ST78	CC88	*cl*	-	*3*	-	-	-	-	-	-	+	-	-	-	-	-	-	+	-	+	-
SM_A247	2012	SSTI	Gemany/Thailand[2]	t2526	07-12-21-17-13-13-13-34-33-13	ND	CC88	*p*	-	*3*	-	-	+	-	-	+	+	-	-	-	-	-	+	+	+	+	+
SM_A249	2012	SSTI	Gemany/Thailand[2]	t2526	07-12-21-17-13-13-13-34-33-13	ND	CC88	*p*	-	*3*	-	-	+	-	-	+	+	-	-	-	-	-	+	+	+	+	+
FS190	2009	sepsis	Finnland	t3202	07-12-21-17-13-13-34-13-33-34	ND	CC88	-	-	*3*	+	-	-	-	-	-	+	-	-	-	-	-	-	+	-	+	+
Z1162	1995	col	Australia	t3205	07-12-21-17-21-34	ST78	CC88	*cl*	-	*3*	-	-	-	+	-	-	+	-	-	-	-	-	-	+	-	+	+
SM_A189	2010	SSTI	Germany	t3341	26-12-21-17-13-34-34-34-33-34	ND	CC88	-	-	*3*	-	-	+	-	-	+	+	-	-	-	-	-	-	+	-	+	-
SM_B45	2005	blood culture	Germany	t4015	07-21-17-34-34-34-34-33-34	ND	CC88	-	-	*3*	-	-	-	-	-	-	+	-	-	-	-	-	-	+	-	+	-
FS208	2009	sepsis	Finnland	t448	07-12-21-17-13-13-34-33-34	ND	CC88	*cl*	-	*3*	-	-	-	-	-	+	+	-	-	-	-	-	-	+	-	+	+
cb220	2012	CRSwNP	Belgium	t690	07-12-21-17-13-13-34-34-34-33-34	ND	CC88	-	-	*3*	-	-	-	-	-	+	+	-	-	-	-	-	-	+	-	+	+
SM_Z1208	2009	unknown	United Arabic Emirates	t690	07-12-21-17-13-13-34-34-34-33-34	ND	CC88	*p kq*	-	*3*	-	-	+	+	+	+	+	-	-	-	-	-	+	+	+	+	+
A7	2007	IV device infection	New Zealand	t692	07-12-21-17-34-34-34-34-33-34	ST78	CC88	-	-	*3*	-	-	-	-	-	-	+	+	-	-	-	-	-	+	-	+	-
SM_A68	2005	SSTI	Germany	t693	07	ND	CC88	*p kq*	-	*3*	-	-	+	-	+	+	+	-	-	-	-	-	+	+	+	+	-
SM_O43	2005	empyema in knee	Germany	t730	07-34-34-34-33-34	ST88	CC88	-	-	*3*	-	-	-	-	-	-	+	-	-	-	-	-	-	+	-	+	-
sh05306	2009	col	Germany	t7413	26-17-66-32-17-23-24	ST88	CC88	-	-	3	-	-	-	-	+	-	-	-	-	-	-	-	-	+	-	+	-
Z1383	2000	unknown	Australia	t786	07-12-21-17-13-34-34-33-34	ND	CC88	*cl*	-	*3*	-	-	-	+	-	-	+	-	-	-	-	-	-	+	-	+	+

[1] *spa* types were clustered by BURP analysis into CCs and corresponding MLST CCs were deduced using the Ridom database.

[2] Isolated in Gemany after recent visit to Thailand

Key: col = nasal colonization (nasopharyngeal sample); SSTI = skin and soft tissue infection; CRSwNP = chronic rhinosinusitis with nasal polyps; *agr* = accessory gene regulator; Staphylococcal enterotoxins (SEs) are indicated by single letters (a = *sea* , etc.). *tst* = toxic shock syndrome toxin 1 gene; *egc* = superantigen genes of the enterotoxin gene cluster, i.e. *seg, sei, sem, sen, seo,* and *seu* ; *eta/etd* = exfoliative toxins a and d; *luk-PV* = Panton-Valentine leukocidine; gene; *Sa1int* = *S. aureus* integrase type 1; *sak* = Staphylokinase gene, chp = gene encoding the chemotaxis inhibitory protein; *scn* = staphylococcal complement inhibitor gene; AmpR = ampicillin resistance
