# Supplementary material for: Laboratory Mice Are Frequently Colonized with Staphylococcus aureus and Mount a Systemic Immune Response—Note of Caution for In vivo Infection Experiments
Source: Front Cell Infect Microbiol. 2017 May 2;7:152. doi: 10.3389/fcimb.2017.00152 (PMC5411432; doi:10.3389/fcimb.2017.00152)
Supplement: Supplementary file 5 [file Table5.PDF]

S5 Table: Genotype, virulence genes, and phage patterns of symptom-free colonized C57BL/6 mice and mice with spontaneous preputial gland adenitis from two Charles River breeding facilities (Kingston and Hollister).

				Spa genotyping			Virulence genes							Phage genes									
Strain ID	Year	Source	Type of infection	*spa* type	*spa* repeats	deduced MLST CC[1]	MGE-encoded SAgs	*egc* SAgs	*agr*	*eta*	*etd*	*pvl*	*mecA*	*Sa1int*	*Sa2int*	*Sa3int*	*Sa4int*	*Sa5int*	*Sa6int*	*Sa7int*	*sak*	*chp*	*scn*
PGA-1	2013	Kingston	PGA	t12341	07-12-596-17-13-34-34-33-34	CC88	-	-	*3*	-	-	-	-	+	-	-	-	-	-	-	-	-	-
PGA-2	2013	Kingston	PGA	t12341	07-12-596-17-13-34-34-33-34	CC88	-	-	*3*	-	-	-	-	+	-	-	-	-	-	-	-	-	-
PGA-3	2013	Kingston	PGA	t12341	07-12-596-17-13-34-34-33-34	CC88	-	-	*3*	-	-	-	-	+	-	-	-	-	-	-	-	-	-
PGA-4	2013	Kingston	PGA	t12342	07-148-596-17-13-34-34-33-34	CC88	-	-	*3*	-	-	-	-	+	-	-	-	-	-	-	-	-	-
PGA-5	2013	Kingston	PGA	t12341	07-12-596-17-13-34-34-33-34	CC88	-	-	*3*	-	-	-	-	+	-	-	-	-	-	-	-	-	-
PGA-6	2013	Kingston	col	t12341	07-12-596-17-13-34-34-33-34	CC88	-	-	*3*	-	-	-	-	+	-	-	-	-	-	-	-	-	-
PGA-7	2013	Kingston	col	t12341	07-12-596-17-13-34-34-33-34	CC88	-	-	*3*	-	-	-	-	+	-	-	-	-	-	-	-	-	-
PGA-8	2013	Kingston	col	t12341	07-12-596-17-13-34-34-33-34	CC88	-	-	*3*	-	-	-	-	+	-	-	-	-	-	-	-	-	-
PGA-9	2013	Kingston	col	t12341	07-12-596-17-13-34-34-33-34	CC88	-	-	*3*	-	-	-	-	+	-	-	-	-	-	-	-	-	-
PGA-10	2013	Kingston	col	t12341	07-12-596-17-13-34-34-33-34	CC88	-	-	*3*	-	-	-	-	+	-	-	-	-	-	-	-	-	-
PGA-11	2013	Hollister	PGA	t693	07	CC1[2]	*h*	-	*3*	-	-	-	-	-	-	-	-	-	-	-	-	-	-
PGA-12	2013	Hollister	PGA	t559	07-23-21-13	CC1	*h*	-	*3*	-	-	-	-	-	-	-	-	-	-	-	-	-	-
PGA-13	2013	Hollister	PGA	t559	07-23-21-13	CC1	*h*	-	*3*	-	-	-	-	-	-	-	-	-	-	-	-	-	-
PGA-14	2013	Hollister	PGA	t9419	17-21-13	CC1	*h*	-	*3*	-	-	-	-	-	-	-	-	-	-	-	-	-	-
PGA-16	2013	Hollister	col	t559	07-23-21-13	CC1[2]	*a h k q*	-	*3*	-	-	-	-	-	-	+	-	-	-	-	-	+	+
PGA-17	2013	Hollister	col	t559	07-23-21-13	CC1	*h*	-	*3*	-	-	-	-	-	-	-	-	-	-	-	-	-	-
PGA-18	2013	Hollister	col	t559	07-23-21-13	CC1[2]	*h*	-	*3*	-	-	-	-	-	-	-	-	-	-	-	-	-	-
PGA-19	2013	Hollister	col	t693	07	CC1	*h*	-	*3*	-	-	-	-	-	-	-	-	-	-	-	-	-	-
PGA-20	2013	Hollister	col	t559	07-23-21-13	CC1	*h*	-	*3*	-	-	-	-	-	-	-	-	-	-	-	-	-	-

1 *spa* types were clustered by BURP analysis into CCs and corresponding MLST CCs were deduced using the Ridom database.
2 MLST typing results: ST1.
Key: col = colonization (nasopharyngeal sample); PGA = preputial gland adenitis (pus sample); *agr* = accessory gene regulator; Staphylococcal enterotoxins (SEs) are indicated by single letters (a = *sea* , etc.). *tst* = toxic shock syndrome toxin 1 gene; *egc* = superantigen genes of the enterotoxin gene cluster, i.e. *seg, sei, sem, sen, seo,* and *seu* ; *eta/etd* = exfoliative toxins a and d; *luk-PV* = Panton-Valentine leukocidine gene; *Sa1int* = *S. aureus* integrase type 1; *sak* = Staphylokinase gene, chp = gene encoding the chemotaxis inhibitory protein; *scn* = staphylococcal complement inhibitor gene
