# Supplementary material for: Laboratory Mice Are Frequently Colonized with Staphylococcus aureus and Mount a Systemic Immune Response—Note of Caution for In vivo Infection Experiments
Source: Front Cell Infect Microbiol. 2017 May 2;7:152. doi: 10.3389/fcimb.2017.00152 (PMC5411432; doi:10.3389/fcimb.2017.00152)
Supplement: Supplementary file 6 [file Table6.PDF]

S6 Table: Binding of murine IgG serum antibodies to recombinant S. aureus proteins using the FlexMAP system.

					Median Fold change[3]				raw data																								
Recombinant protein[1]	Protein name	Gene ID	Strain	Sub-cellular location[2]	Colonized - Kingston	PGA - Kingston	Colonized - Hollister	PGA - Hollister	SOPF	SOPF	SOPF	SOPF	SOPF	CK	CK	CK	CK	CK	CH	CH	CH	CH	CH	AK	AK	AK	AK	AK	AH	AH	AH	AH	AH
Bifunctional autolysin (Endo-beta-N-acetylglucosaminidase) GL domain	Atl	SAOUHSC_00994	NCTC8325	ec	3.2 *	9.7 **	6.3 *	2980.1 *	2839	14274	5691	5588	5806	5883	18252	33355	6836	143531	97708	6912	35919	32576	1450047	1231250	47384	55000	36198	373449	19164228	18877095	16961928	13664329	8939
Bifunctional autolysin (N-acetylmuramoyl-L-alanine amidase) AM domain	Atl	SAOUHSC_00994	NCTC8325	ec	0.3	1.5	0.5	2.5	393	227	633	‡	‡	471	112	96	432	109	‡	193	121	192	13368	‡	367	598	1004	577	72312	998	362	10419	676
Isocitrate dehydrogenase [NADP]	CitC	SACOL1741	COL	ic	1.7	2.2	4.4	2.7	126	127	295	325	176	141	417	303	12672	188	210	802	‡	757	1192	235	381	229	802	452	261	657	450	506	‡
Clumping factor A#	ClfA	SAOUHSC_00812	NCTC8325	cw	NA	3.1 *	1.5	144.8	59	‡	126	157	125	‡	‡	‡	‡	‡	‡	130	101	248	7901	4227	173	229	382	1493	19486	33106	458	18136	113
Clumping factor B#	ClfB	SAOUHSC_02963	NCTC8325	cw	1.8	8.0	4.7	4.1	‡	501	430	‡	4748	‡	659	‡	‡	1191	2347	428	2490	1109	7518	9709	‡	‡	1394	4025	5203	85329	2075	968	772
Collagen adhesin domain 1#	Cna	Nr1_BAF45800	NN6	cw	1.3	0.9	2.4	4.4	63	51	434	345	63	106	75	151	82	38	120	164	60	222	153	47	52	76	287	55	278	79	127	3277	735
Collagen adhesin domain 2#	Cna	Nr2_BAF45800	NN6	cw	1.6	2.3	2.1	3.6	18	‡	‡	119	40	64	21	71	‡	‡	41	85	36	164	214	67	193	118	90	67	143	41	65	528	199
Coagulase	Coa	SAOUHSC_00192	NCTC8325	ec	0.6	0.7	0.7	0.9	‡	1105	5444	677	‡	401	942	‡	3488	408	295	587	1184	1514	813	539	431	1469	2212	791	871	‡	976	‡	1599
Fibrinogen-binding protein	Efb	SAOUHSC_01114	NCTC8325	ec	1.1	3.0 **	1.2	3.8 **	2338	5572	4111	6029	2407	4215	4876	4341	3912	5361	5085	4947	5943	4917	12154	11295	7033	17088	48059	12491	23471	15421	16308	10581	10835
Extracellular matrix protein-binding protein	Emp	SAOUHSC_00816	NCTC8325	cw	2.9 *	4.1 *	1.6	1.5	252	632	371	603	‡	1425	4583	862	973	3900	663	787	521	796	1684	1982	5160	1940	1737	2936	1706	613	880	736	376
Uncharacterized protein, ESAT-6 family	ESAT-6	SAOUHSC_00257	NCTC8325	ec	1.5	4.2	2.1	2.1	198	105	‡	240	‡	301	300	323	‡	‡	‡	246	352	893	470	‡	245	‡	1410	‡	636	240	‡	364	484
3-hydroxyacyl-CoA dehydrogenase protein	FadB	SAOUHSC_00196	NCTC8325	ic	1.7	1.7	2.5	1.4	623	901	994	655	2157	1189	1513	1623	6227	1475	2309	1150	914	2300	2727	1537	1288	2037	7090	872	1273	762	1080	3852	2816
Glycerol ester hydrolase	Geh	SAUSA300_0320	USA300	ec	0.3	0.7	2.3	47.9	‡	‡	‡	216	45	35	‡	‡	‡	‡	‡	‡	‡	‡	297	157	‡	‡	‡	38	22910	6265	6385	4604	1739
Glycerophosphoryl diester phosphodiesterase	GlpQ	SAUSA300_0862	USA300	ec	0.6 *	1.6	1.2	2.0	131	1168	193	263	147	89	85	130	122	187	1129	649	227	220	217	289	307	282	1027	359	266	381	429	762	361
Transcription elongation factor	GreA	SACOL1665	COL	ic	1.3	1.0	3.2 **	2.6 *	310	170	264	306	191	372	2131	353	274	252	835	1171	406	848	2678	230	277	201	2309	‡	2124	968	687	301	452
GroEL protein	GroEL	SAUSA300_1982	USA300	ic	0.9	1.0	0.7	1.5	50	‡	20	25	40	42	‡	‡	15	‡	22	‡	‡	‡	‡	‡	32	‡	‡	‡	84	45	‡	54	45
Alphahemolysin#	Hla	SAOUHSC_01121	NCTC8325	ec	2.6	3.0	1.1	5.0	377	‡	1044	1204	407	1905	1224	2723	3453	711	1248	789	623	698	1517	1289	1034	10719	2178	2512	1662	‡	852	6995	5650
Betahemolysin	Hlb	SACOL2003	COL	ec	4.0 *	12.3 **	11.9 **	680.1 **	77	52	77	175	143	136	281	309	339	463	300	919	841	3333	20778	1640	413	2654	647	954	521166	123077	52616	34451	1041
Gamma-hemolysin component B	HlgB	SACOL2422	COL	ec	2.2 **	4.8 **	4.2	19.9 **	11	9	12	14	12	16	34	26	35	16	10	16	128	50	81	37	57	229	56	54	86691	122	3062	235	60
Gamma-hemolysin component C	HlgC	SACOL2421	COL	ec	2.2 *	7.1 **	2.8	383.3 **	91	83	145	129	68	168	133	284	198	205	288	252	96	105	384	161	2029	645	685	256	85496	3190	34879	30658	197223
Immunodominant staphylococcal antigen A	IsaA	SAOUHSC_02887	NCTC8325	ec	0.8	1.0	2.7	556.8	713	527	954	223	258	252	747	399	2939	333	637	281	1647	1422	2518	1134	515	507	979	261	305530	293391	339	510839	283170
Immunodominant staphylococcal antigen B	IsaB	SAOUHSC_02972	NCTC8325	ec	0.4 *	1.1	1.3	1.5	320	1155	763	453	217	198	308	165	158	269	569	652	314	474	893	305	159	549	1975	517	662	17238	644	701	1427
Iron-regulated surface determinant protein A#	IsdA	SAOUHSC_01081	NCTC8325	cw	1.1	1.6	1.0	102.9	‡	‡	112	90	‡	‡	193	46	‡	114	129	83	26	296	105	362	‡	23	149	166	410327	10374	67416	81	133
Iron-regulated surface determinant protein B#	IsdB	SAOUHSC_01079	NCTC8325	cw	33.7	43.7	1.7	2183.6	‡	‡	‡	‡	64	678	2147	1969	2971	26768	‡	‡	32	110	1241	8336	2209	2784	513	6167	1977975	138991	2912837	13247	197
Triacylglycerol lipase	Lip	SAUSA300_2603	USA300	ec	2.0	0.9	0.9	2.8	‡	25	‡	‡	‡	‡	‡	88	13	‡	‡	‡	‡	25	22	57	28	15	‡	18	883	163	71	24	27
MHC class II analog protein	Map	SAOUHSC_02161	NCTC8325	mem	1.0	2.8	9.6	2327.1	‡	‡	770	244	‡	‡	‡	220	203655	518	8700	398	18164	852	4881	1437	1384	4824	583	2388	729787	795906	1433494	3784202	1179196
Peptide methionine sulfoxide reductase	MsrA2	SAOUHSC_01432	NCTC8325	unkn	1.2	1.8 *	1.4	1.6 **	1237	1204	654	1450	809	1263	1413	1247	2211	4677	2596	1225	1204	1636	2848	1442	7156	2182	9516	1789	1817	4263	1562	1912	1967
Peptide methionine sulfoxide reductase	MsrB	SAOUHSC_01431	NCTC8325	ic	1.7	2.0	1.6	3.1	113	1116	93	192	67	93	191	374	227	135	‡	178	‡	141	413	216	294	156	2585	231	356	210	526	1040	244
Thermonuclease	Nuc	SAOUHSC_01316	NCTC8325	ec	0.5	2.4	1.6	1.2	2100	3736	8715	16734	58696	2727	4353	19909	11606	3577	13570	70694	6845	1904	20773	1314	3817	20807	102828	21981	10834	23666	11473	9632	7130
Penicillin-binding protein 2	Pbp2	SAOUHSC_01467	NCTC8325	mem	1.2	2.0	2.8	1.9	886	1060	2448	1938	965	1218	2651	1227	2486	1038	2979	1179	4965	3030	2430	2825	‡	1418	5323	1130	7304	2042	977	6901	740
Serine/threonine-protein kinase	PknB	SA1063	N315	ic	2.8	3.7 *	4.2 *	8.1 **	53	153	77	181	51	131	220	90	252	239	470	513	206	163	324	286	96	661	536	211	627	413	344	3334	1776
Phospholipase C	Plc	SAUSA300_0099	USA300	ec	102.2	630.4	237.5	8750.0	‡	2	1	‡	‡	179	193	35	271	34	417	1328	274	138	1858	2847	165	1107	7	26218	39478	15049	75950	9034	9481
Foldase protein	PrsA	SAOUHSC_01972	NCTC8325	mem	0.9	0.5	2.5	18.7	‡	‡	288	242	‡	61	‡	239	31811	‡	886	173	‡	1395	424	105	‡	280	‡	140	31623	4072	254	‡	5829
Adenylosuccinate synthetase	PurA	SACOL0018	COL	ic	1.3	2.0	1.2	1.4	3078	5183	7404	4337	3700	4580	7559	3963	25821	5572	6191	4817	3639	5393	11981	6742	4885	8658	27646	9100	23751	5735	6550	3215	6202
Uncharacterized protein	SACOL0444	SACOL0444	COL	unkn	0.9	2.0 *	1.4	3.5	47	46	46	66	44	38	84	42	‡	41	50	33	‡	87	77	76	51	‡	2038	111	827	299	46	159	39
Uncharacterized protein	SACOL0480	SACOL0480	COL	unkn	0.0	0.6	0.1	0.1	104	3	95	‡	‡	‡	‡	4	4	4	4	5	‡	‡	9	‡	‡	‡	15	104	70	8	11	43	9
Surface protein, putative	SACOL0985	SACOL0985	COL	mem	0.7	0.7	0.5	1.3	80	28	34	46	23	17	133	17	23	28	13	16	15	47	140	45	14	17	116	23	33	66	4512	43	25
Uncharacterized protein	SACOL1788	SACOL1788	COL	cw	1.8	2.7	1.0	1.1	‡	‡	196	156	‡	‡	469	‡	‡	162	191	174	‡	155	256	‡	‡	475	534	157	436	174	190	206	142
Uncharacterized protein	SACOL1802	SACOL1802	COL	unkn	0.8	3.8	0.9	3.7	19	21	15	10	14	12	9	12	17	14	9	13	11	22	28	46	4	‡	90	63	27	103	97	53	10
Surface protein, putative	SACOL2197	SACOL2197	COL	mem	0.7	1.0	1.1	2.6	‡	‡	‡	3	‡	1	‡	3	‡	‡	2	3	2	4	4	‡	2	‡	7	3	7	8	7	48	7
Truncated MHC class II analog protein	SAOUHSC_02466	SAOUHSC_02466	NCTC8325	mem	1.0	1.4	1.2	2.2 *	56	67	79	53	48	30	95	44	57	63	54	79	54	109	66	49	55	181	80	88	121	94	121	150	79
Serine-aspartate repeat-containing protein D domain 1#	SdrD	SAOUHSC_00545	NCTC8325	cw	0.8	1.2	1.9	17.0	147	102	‡	190	‡	‡	138	68	119	111	92	666	97	282	305	66	‡	‡	716	178	7089	‡	142	4875	106
Serine-aspartate repeat-containing protein D domain 2#	SdrD	SAOUHSC_00545	NCTC8325	cw	NA	NA	NA	NA	‡	‡	‡	‡	‡	‡	‡	‡	‡	2873	4225	868	‡	2251	16274	27337	‡	‡	3163	9727	42478	202154	11346	1509	1547
Serine-aspartate repeat-containing protein E#	SdrE	SAOV0598	ED133	cw	NA	1.4	2.3	18.1	‡	292	234	137	‡	‡	‡	‡	‡	‡	‡	137	611	449	1581	2773	338	157	312	‡	10446	7924	572	‡	98
Serine-aspartate repeat protein G#	SdrG	ISK_1994	*S. epidermidis* IS-K	cw	0.9	2.1	3.2	1.2	‡	170	698	‡	‡	‡	‡	‡	‡	375	‡	‡	114	‡	2623	4158	‡	393	482	1323	961	7646	512	442	332
Superoxide dismutase	SodA	SAOUHSC_01653	NCTC8325	ec	2.3	1.7 *	2.1	3.2 *	25	58	39	56	38	97	83	139	‡	47	82	25	47	120	144	58	118	65	‡	69	125	47	126	132	88
Serine protease A	SplA	SACOL1869	COL	ec	0.9	4.6	0.7	3.4 **	11	7	7	10	9	9	7	8	33	14	7	6	4	16	40	42	5	‡	60	44	31	69	12	43	18
Serine protease B	SplB	SAOUHSC_01941	NCTC8325	ec	1.0	3.8	2.9	71.1 **	61	106	200	440	75	97	96	445	640	102	311	8634	112	276	658	1989	69	194	404	5162	12690	6333	36735	7550	5940
Serine protease D	SplD	SAUSA300_1755	USA300	ec	3.5	291.5	13.9 *	5332.5 *	7	4	35	3	4	5	32	9	15	153	10	3066	60	29	65	2452	5	‡	51	38850	22903	79959	123138	16	1720
Staphylococcal secretory antigen	SsaA2	SAOUHSC_02571	NCTC8325	ec	0.6	0.7	1.4	2.7	113	270	7626	600	226	166	116	329	1484	157	320	440	147	639	365	333	146	161	1100	200	584	481	3572	736	934
Staphopain B	SspB	SACOL1056	COL	ec	1.3	1.0	1.3	43.0	‡	‡	28	‡	23	‡	‡	‡	33	‡	33	‡	‡	‡	‡	26	‡	‡	84	26	‡	‡	‡	1088	‡
Trigger factor	Tig	SACOL1722	COL	ic	1.1	3.6	2.2	3.8	245	‡	626	201	‡	230	862	324	207	281	266	1651	‡	‡	551	‡	1919	404	1273	485	2402	‡	1494	353	268
Elongation factor Tu	Tuf	SA0506	N315	ic	3.3	2.7	0.9	1.4	1446	‡	‡	8569	‡	29433	2788	‡	41701	3292	3091	‡	2130	6070	6120	13463	2518	8758	487683	14329	7045	9892	5717	31989	2898
DNA segregation ATPase and related proteins domain 2	YukA	SAOUHSC_00262	NCTC8325	mem	1.1	1.0	1.0	0.7	‡	1218	1110	2966	‡	‡	1298	‡	1320	844	1237	1059	663	1248	1845	884	2272	1204	2596	960	4545	2213	862	578	671
DNA segregation ATPase and related proteins domain 1	YukA	SAOUHSC_00262	NCTC8325	mem	0.7	1.3	1.5	0.7	889	5757	941	894	560	825	625	648	864	384	1724	372	761	1351	2168	1222	549	1176	2630	858	602	369	1376	415	836

1 Vaccine candidates previously or currently in clinical studies are labelled with #.

2 The subcellular location was predicted using PSORTb version 3.0.2 .

3 The fold change in serum IgG titers in exposed (colonized or infected) mice was calculated by deviding the median MFI signal of the exposed mice (n=5) by the median MFI of SOPF mice (n=5). Two-tailed Mann Whitney U test. * = p<0.05, ** = p<0.01

Key: ec, extracellular; mem, membrane; cw, cell wall; ic, cytoplasm/intracellular; unkn, unknown; NA, not applicable; ‡, measured MFI values were below detection limit.
