# Supplementary figures and images for: Laboratory Mice Are Frequently Colonized with Staphylococcus aureus and Mount a Systemic Immune Response—Note of Caution for In vivo Infection Experiments
Source: Front Cell Infect Microbiol. 2017 May 2;7:152. doi: 10.3389/fcimb.2017.00152 (PMC5411432; doi:10.3389/fcimb.2017.00152)

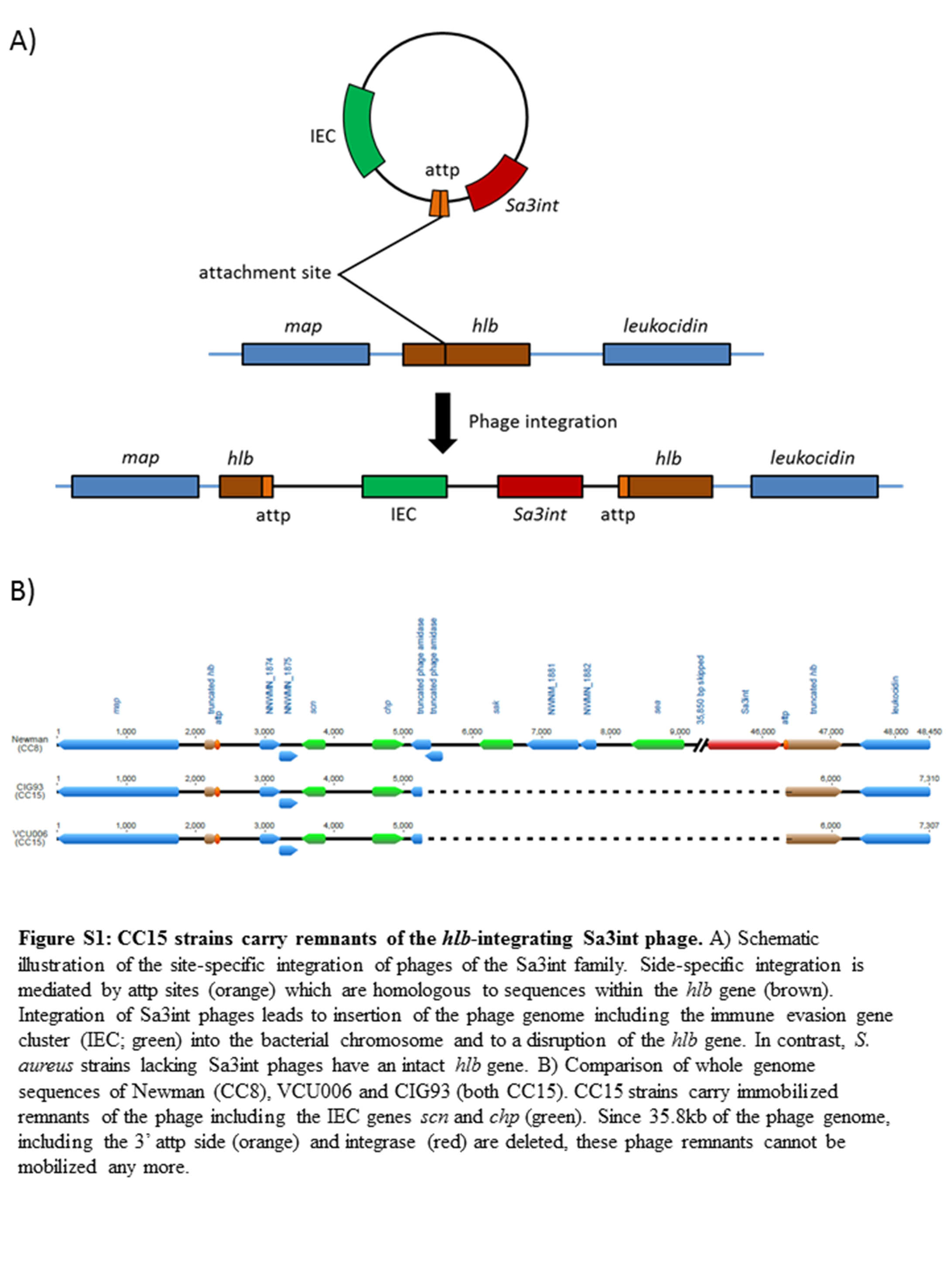

Supplement: Supplementary file 7 [file Image1.TIF]

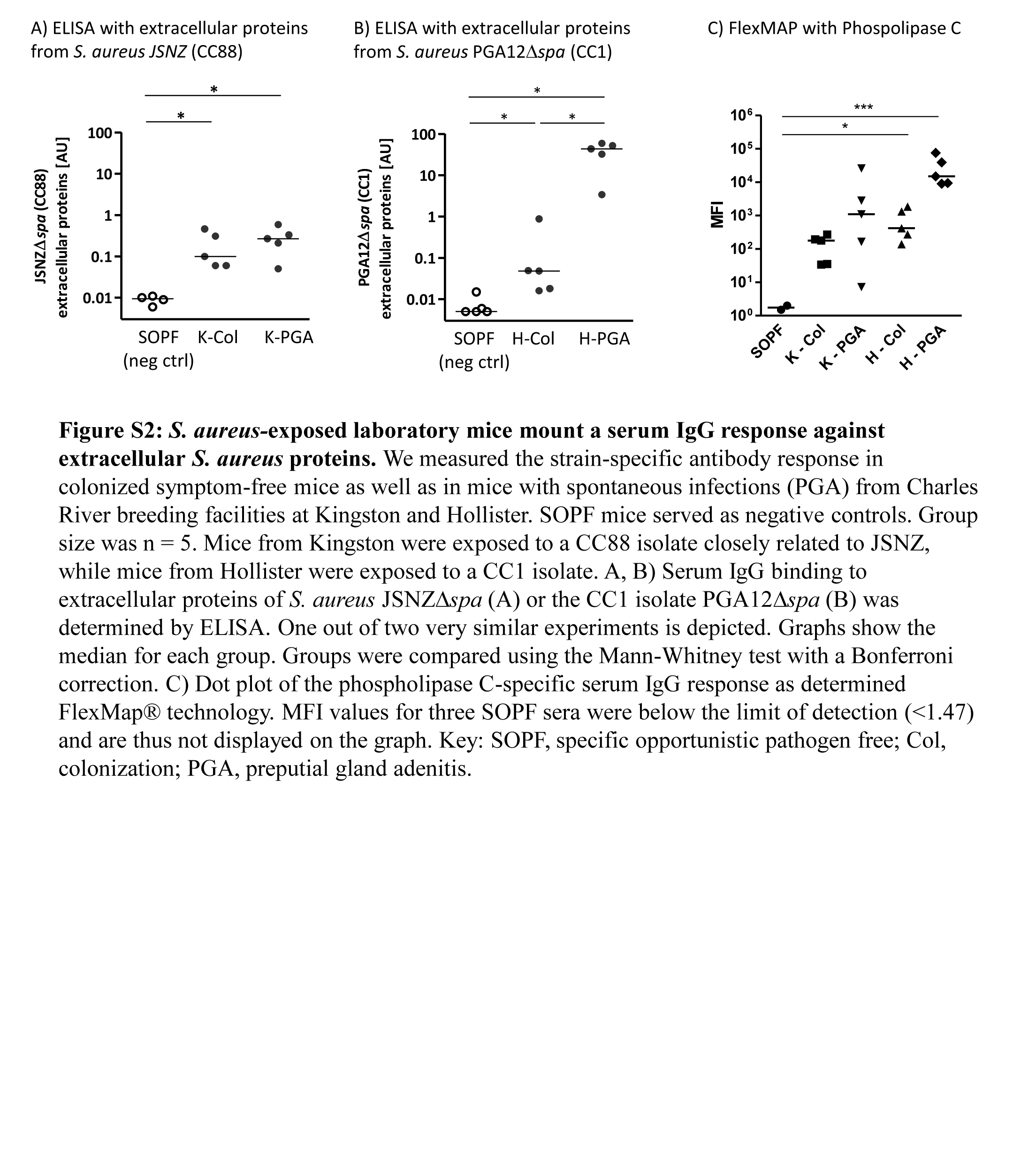

Supplement: Supplementary file 8 [file Image2.TIF]
